# TLR8 Stimulation Leads to Microenvironment Activation and Enhanced Antibody-Dependent Cellular Responses in CLL

**DOI:** 10.3390/ijms27188003

**Published:** 2026-09-09

**Authors:** Giovanna Merchand-Reyes, Gauri Anyarambhatla, Maria L. Valencia Pena, Renie Mensah-Bonsu, Ramasamy Santhanam, Xiaokui Mo, Frank H. Robledo-Avila, Susheela Tridandapani, Jonathan P. Butchar

**Affiliations:** 1 Department of Internal Medicine, College of Medicine, The Ohio State University Wexner Medical Center, Columbus, OH 43210, USA; 2 Department of Radiation Oncology, College of Medicine, The Ohio State University Wexner Medical Center, Columbus, OH 43210, USA; 3 Center for Biostatistics, College of Medicine, The Ohio State University Wexner Medical Center, Columbus, OH 43210, USA; 4 Department of Biomedical Informatics, College of Medicine, The Ohio State University Wexner Medical Center, Columbus, OH 43210, USA; 5 Center for Microbe and Immunity Research, Abigail Wexner Research Institute, Nationwide Children’s Hospital, Columbus, OH 43205, USA; frank.robledoavila@nationwidechildrens.org

**Keywords:** Toll-like receptors, Fc receptors, monocytes, NK cells, neutrophils, innate immunity

## Abstract

Toll-like receptors (TLRs) are crucial for eliciting immune responses against pathogens such as bacteria and viruses. TLR activation has also been studied for its potential to drive antitumor activity in immune cells. TLR8 is an intracellular receptor that recognizes single-stranded RNA and promotes antiviral responses, including the release of cytokines such as TNFα and type I IFN. Previously we have shown that TLR8 activation can also modulate the expression of Fcγ receptors (FcγR), which are major drivers of antibody-mediated tumor cell clearance. Hence, TLR8 agonist treatment represents a potential means of overcoming tumor-related immunosuppression and enhancing the efficacy of antibody therapy. In chronic lymphocytic leukemia, the use of therapeutic antibodies against CD20 has become standard for treatment. However, the immune effector cells show suppressed phenotypes and weakened responses compared to their healthy-donor counterparts. Here, we investigated TLR8 agonist treatment as a means to overcome this immune suppression and enhance cellular antibody-mediated responses. Upon stimulation with the agonist TL8-506, primary CLL-patient monocytes showed an increase in the expression of activating FcγR in conjunction with reduced expression of regulatory receptors. Cytokine production was also enhanced and there was a shift in phenotypic markers, suggesting the adoption of a more M1-like state. Treatment with TLR8 agonist also elicited production of IFNγ and Granzyme B and enhanced antibody-dependent cellular cytotoxicity of antibody-coated B cells by NK cells. Agonist treatment also increased the phagocytic activity of neutrophils but did not affect the formation of neutrophil extracellular traps (NETs). Taken together, these results suggest that TLR8 agonists may help counteract the suppressed phenotypes of immune effector cells and may enhance the efficacy of antibody therapy for CLL.

## 1. Introduction

Chronic lymphocytic leukemia (CLL) is a hematologic malignancy wherein there is excessive survival and proliferation of CD5^+^ B cells [1]. It is the leading type of leukemia, typically diagnosed at a median age of 70 years [1]. Although it is generally well-managed, relapse remains problematic, with over 35% of patients above 75 years of age requiring second-line treatment at a median of 22 months [2]. Along with this, up to 10% of all diagnosed CLL patients undergo a change to a more aggressive disease such as diffuse large B-cell lymphoma (DLBCL), known as Richter’s transformation [3]. Hence, there is a continued need for new therapies.

One commonly employed treatment for CLL is antibody therapy. Approved antibodies consist primarily of chimeric or engineered monoclonal antibodies directed against CD20, which is selectively expressed on B/CLL cells [1]. Currently, the afucosylated type II anti-CD20 antibody obinutuzumab is routinely used as a standalone agent and in combination with the BCL-2 inhibitor venetoclax or the BTK inhibitors ibrutinib and acalabrutinib [4,5,6]. Antibody therapy against CD20-expressing target cells is mediated in large part by Fcγ receptors (FcγR) [7]. Here, the Fc portions of therapeutic antibodies bind to Fcγ receptors (FcγR) on immune effector cells, triggering responses such as antibody-dependent cellular cytotoxicity (ADCC) and antibody-dependent cellular phagocytosis (ADCP) [8,9,10]. However, the innate immune effector cells mediating these responses are functioning in a suppressed, suboptimal state [11].

It has been well established that a supportive microenvironment is a major factor in CLL [1]. Here, multiple cell types contribute toward CLL-cell support including macrophages, T cells, NK cells and stromal cells [12]. The contribution of neutrophils has also received attention [13]. Within this CLL microenvironment, macrophages represent a major supportive component. These cells, termed nurse-like cells (NLCs) [14,15], promote CLL-cell survival through the expression of factors such as BAFF, APRIL and SDF-1 [14,16]. They also have been shown to promote resistance to antitumor agents such as ibrutinib [17]. Their earlier-stage CD14^+^ counterparts, monocytes, also demonstrate phenotypic and functional signs of immunosuppression including reduced phagocytosis and cytokine production [18,19].

Natural killer (NK) cells are another widely studied innate immune cell type in CLL. They are major effectors of antibody-dependent cellular cytotoxicity (ADCC) against antibody-coated tumor targets and have been shown to orchestrate responses to the anti-CD20 antibody obinutuzumab [20,21]. These cells also cells play a critical role in priming monocytes/macrophages via IFNγ release [22]. Nevertheless, they appear impaired within the setting of CLL, with a defective phenotype and subdued responses [23,24] (and reviewed in [25,26]). This appears to be caused by both cellular and soluble factors [25].

Neutrophils have also been studied for their potential to either exacerbate or potentially combat CLL [13,27]. It has been reported that these cells present an altered and activated phenotype, which involves an increased expression of both CD64 and CD54 compared to age-matched healthy controls (particularly in active disease), as well as enhanced ROS production yet a muted cytokine response to LPS [28]. In addition, CLL-patient-derived neutrophils show impaired killing activity against bacteria [29]. Furthermore, these cells are widely known for their ability to form neutrophil extracellular traps (NETs) [30] (and reviewed in [31,32]). However, this may serve a pro-tumoral role within CLL, resulting in apoptosis resistance and enhanced expression of the activation markers CD86 and CD69 [33]. Finally, BAFF and APRIL are increased in neutrophils from the spleens of Eμ-TCL1 mice (a common CLL model [34]), suggesting pro-leukemic behavior [35].

Given the suppressed nature of these and other cells within the CLL microenvironment, strategies to return them to normal function have been actively pursued. Among these, immune modulators have long been studied for their potential to reverse tumor-related immunosuppression and to enable immune effector cells to regain their ability to engage and destroy the tumor cells [36]. The idea of using such modulators in conjunction with standard treatments is also being explored (reviewed in [1]).

In a previous study, we found that the TLR8 agonist treatment could induce strong cytokine production, as well as Granzyme B release, in primary monocytes [37], suggesting that it may be serve as a powerful agent within the suppressed setting of CLL. In a subsequent study we screened TLR agonists for their effects on FcγR expression and found that agonists for TLR4 and TLR8 were highly effective, especially with regard to downregulating the inhibitory FcγRIIb [38].

Here, based on these earlier findings, we examined whether treatment of CLL-patient immune effector cells with a Toll-like receptor 8 (TLR8) agonist could help reverse their suppressed state and enhance their effectiveness against antibody-coated target cells. We found that treatment with the TLR8 agonist TL8-506 led to changes in FcγR expression and in cellular phenotypes. Agonist treatment also enhanced responses to antibody-coated target cells. Importantly, TLR8 agonist treatment did not increase NET formation, which has been reported to have pro-tumoral effects. Hence, TLR8 agonist treatment may represent a means of activating CLL-associated immune effector cells and enhancing antitumor responses in conjunction with antibody therapy and perhaps other modalities.

## 2. Results

### 2.1. TLR8 Agonists Reduce the Expression of Checkpoint Molecules While Enhancing Antibody-Mediated Cytokine Production

**Figure 1:**
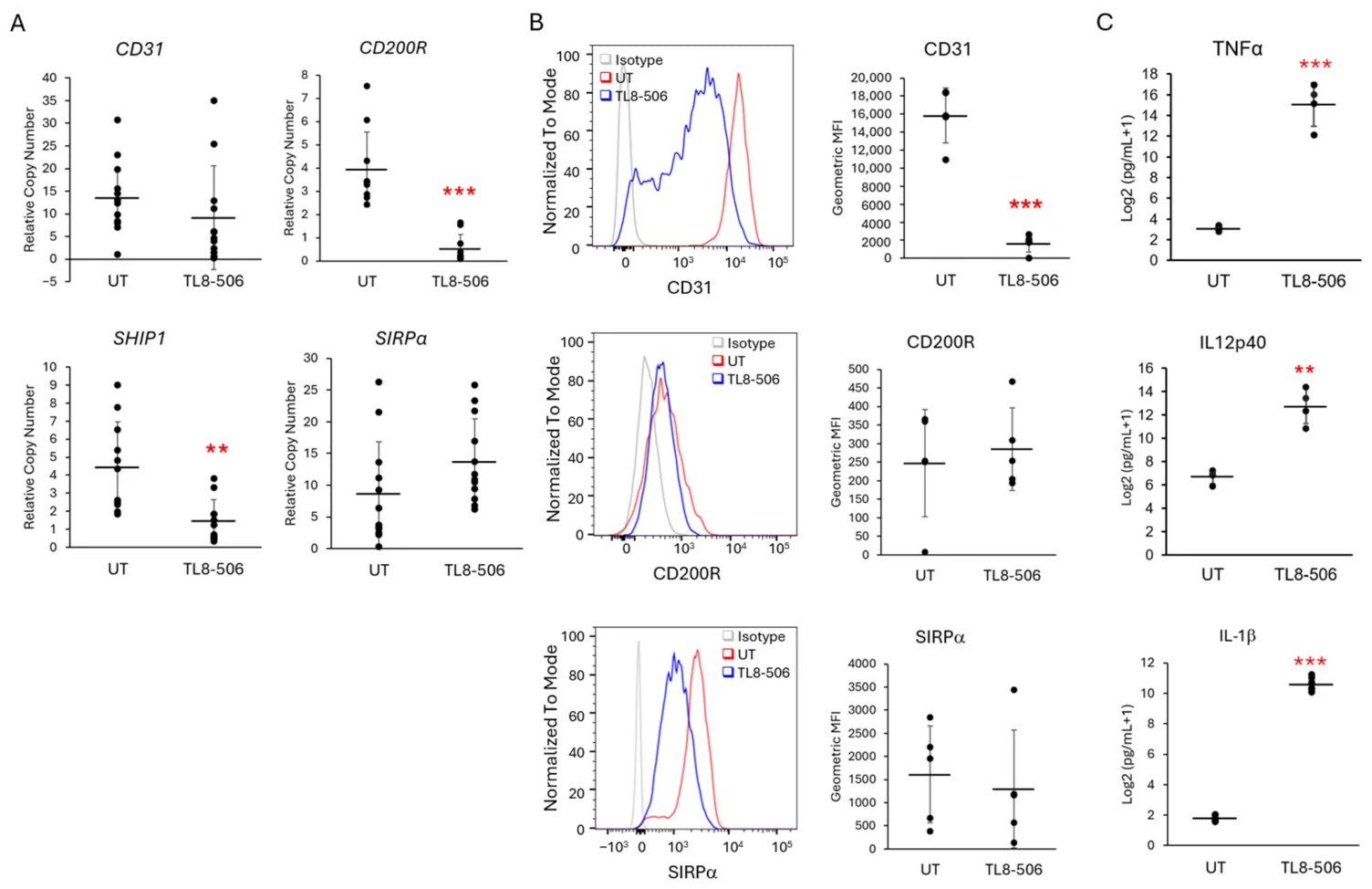
TLR8 agonists reduce expression of regulatory molecules, inducing cytokine release in CLL-patient monocytes. Monocytes from CLL patients were stimulated for 48 h with 1 μM TL8-506. (**A**) Expression of checkpoint molecules *CD31*, *SHIP1*, *CD200R* and *SIRPα* was measured using qPCR (n = 12 for *CD31* and *SIRPα*, n = 11 for *SHIP1* and n = 10 for *CD200R*). (**B**) Surface expression of CD31, CD200R and SIRPα was measured using flow cytometry (n = 5); a representative histogram is shown. (**C**) ELISAs were done with cleared supernatants to measure TNFα (n = 4), IL-12 (p40) (n = 4) and IL-1β (n = 6). ** *p* ≤ 0.01; *** *p* ≤ 0.001.

As discussed above, monocytes from CLL patients show signs of immunosuppression including reduced phagocytosis and cytokine production [18,19]. Here, we tested whether treatment with a TLR8 agonist could help counteract this. We isolated monocytes from peripheral blood of CLL patients and treated them for 48 h with or without 1 μM of the TLR8 agonist TL8-506. Following this, we measured expression of the negative regulatory molecules *CD31* [39], *CD200R* [40], *SHIP1* [41], *SHIP2* [42] and *SIRPα* [43] expression using qPCR. Results showed significant downregulation of *CD200R* and *SHIP1*, with a nonsignificant upregulation of *SIRPα* (Figure 1A). There was a trend toward downregulation of *CD31* that did not reach significance. Expression of *SHIP2* remained unchanged (Supplementary Figure S1). We then repeated this experiment, measuring surface expression of checkpoint molecules using flow cytometry. Results showed a substantial and significant downregulation of CD31 but no change in CD200R (Figure 1B, top and middle panels respectively). Of note, SIRPα fluorescence was reduced in 4 out of the 5 patient samples tested, but this downregulation did not reach significance (Figure 1B, lower panel). We then isolated monocytes and treated them with or without TL8-506 for 48 h and measured levels of TNFα, IL-12 p40 and IL-1β in cleared supernatants using ELISAs. Results showed strongly and significantly upregulated production of all three cytokines, which was consistent across all patient samples tested; of note, two samples tested for TNFα production gave values over the standard curve upon TL8-506 stimulation even after dilution. These results suggest that despite their suppressed state, CLL-patient monocytes can respond strongly to TLR8 agonists.

### 2.2. TLR8 Agonists Modulate FcγR Expression in CLL-Patient Monocytes

Monocytes within the setting of CLL show attenuated FcγR responses [18], so here we tested whether TLR8 agonist treatment could reverse this. Monocytes were enriched from CLL-patient peripheral blood and treated for 48 h with or without TL8-506. Expression of *FcγR* was measured using qPCR.

**Figure 2:**
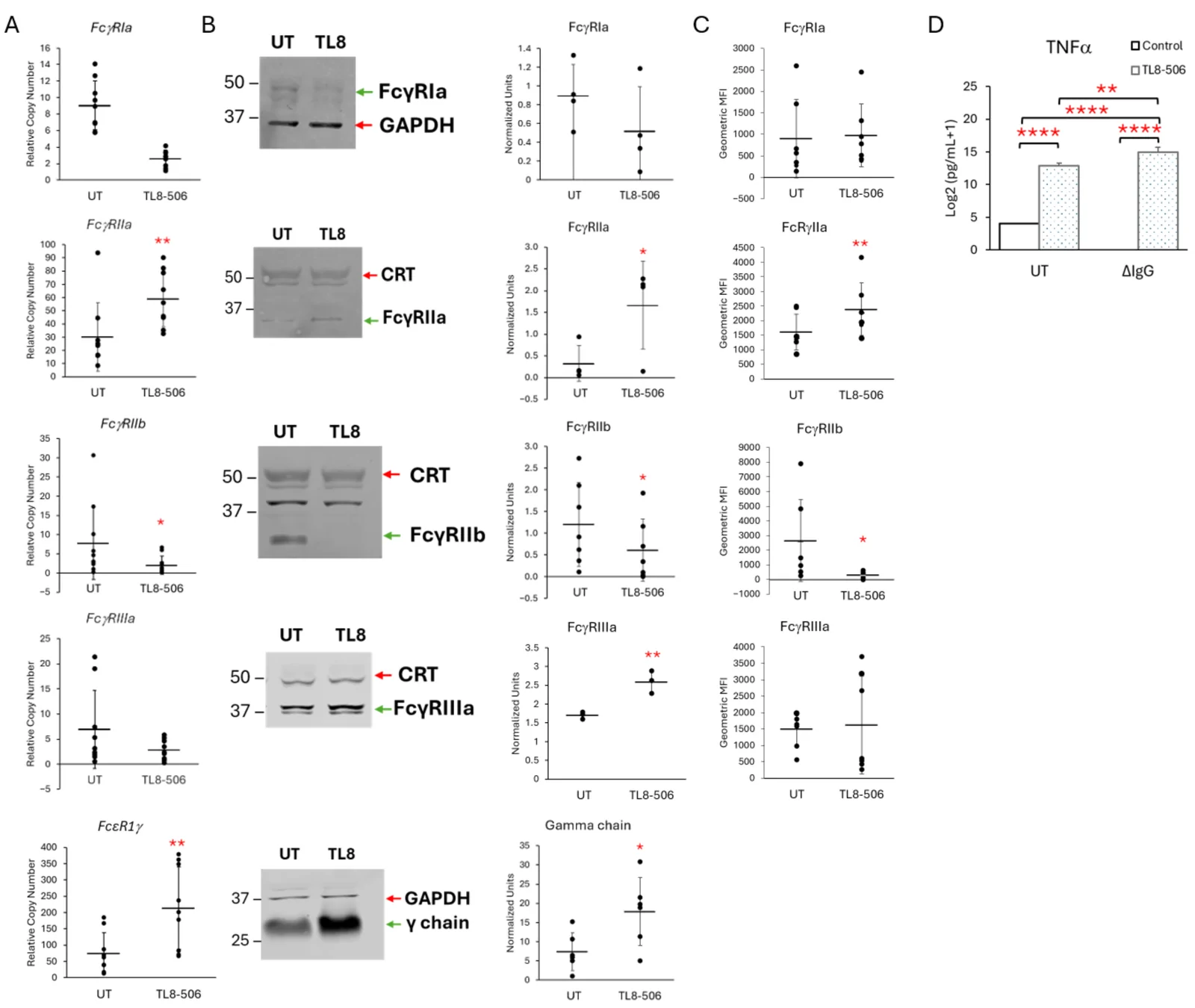
TLR8 agonists modulate FcγR expression in CLL-patient monocytes. Monocytes were isolated from CLL-patient blood and treated with or without 1 μM TL8-506. (**A**) After 48 h, FcγR expression was measured by qPCR (n = 10 for *FcγRIIb*, n = 9 for the others). (**B**) After 48 h, protein levels were measured by immunoblotting; both a representative blot, as well as densitometry (n = 4 for FcyRIA and FcyRIIA, n = 3 for FcyRIIIa, n = 7 for FcyRIIb and n = 6 for the gamma chain) is shown. (**C**) After 48 h, FcγR surface expression was measured by flow cytometry (n = 7). (**D**) Isolated patient monocytes were treated with or without 1 μM TL8-506 for 24 h, then incubated with or without heat-aggregated IgG (ΔIgG) for an additional 24 h. Cleared supernatants were collected and TNFα was measured using ELISA (n = 3). * *p* ≤ 0.05; ** *p* ≤ 0.01; **** *p* ≤ 0.00001. Full-membrane images are shown in Supplementary Figure S2.

Results showed a significant upregulation of *FcγRIIa* and of the gamma chain *FcεRI*γ, as well as a downregulation of *FcγRIIb* (Figure 2A). Similarly, immunoblotting was done to measure protein levels of FcγR. Results showed upregulation of FcγRIIa, FcγRIIIa and the gamma chain, with a downregulation of the inhibitory receptor FcγRIIb (Figure 2B). Blotting also showed a nonsignificant decrease in FcγRIa, consistent with our observations at the transcriptional level.

To gain a clearer understanding of FcγR changes, we also treated the cells for 48 h as above and measured surface expression of FcγR using flow cytometry. Results showed significantly increased FcγRIIa expression and significantly decreased FcγRIIb expression, with no significant changes in FcγRIa or in FcγRIIIa (Figure 2C). Because surface expression of the activating FcγRIIa was increased and that of the inhibitory FcγRIIb was decreased, we predicted that monocyte FcγR function would be enhanced. To test this, we incubated patient monocytes with or without TL8-506 for 24 h, then incubated for 24 h with or without heat-aggregated IgG to cluster the FcγR. Following this, cleared supernatants were collected and TNFα was measured by ELISA. Results showed that TL8-506 significantly enhanced IgG-mediated TNFα production (Figure 2D). Collectively, these results suggest that TLR8 agonist treatment enhances CLL-patient monocyte FcγR expression and function.

### 2.3. TLR8 Agonists Help Overcome the Immunosuppressive Environment

**Figure 3:**
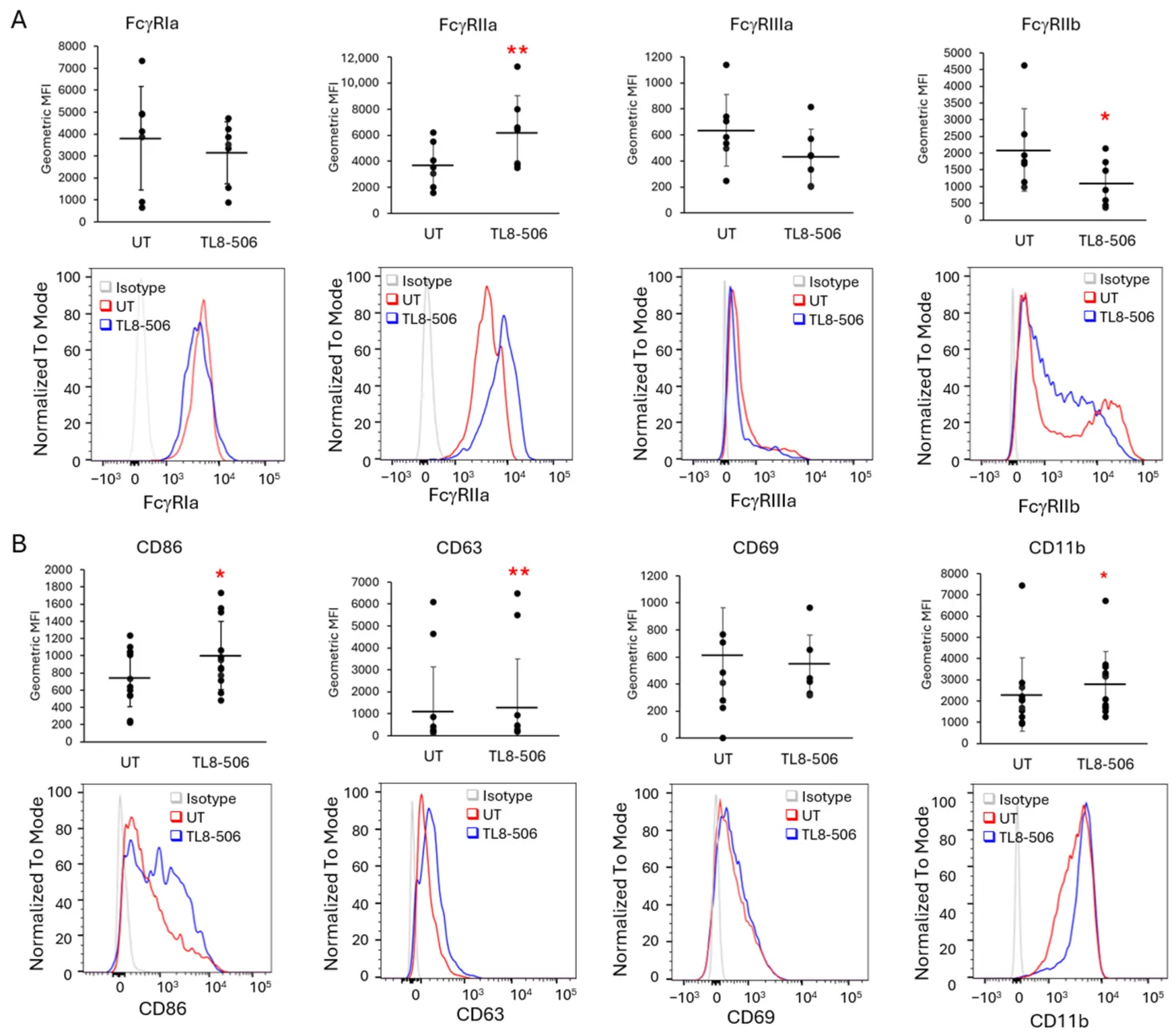
TLR8 agonists help overcome the immunosuppressive environment. WBCs from CLL patients were obtained and incubated with or without 1 μM TL8-506 for 48 h. Expression of (**A**) FcγR (n = 7) and (**B**) activation markers (n = 12) was measured in CD14^+^ cells using flow cytometry. * *p* ≤ 0.05, ** *p* ≤ 0.01.

The above experiments were conducted using isolated monocytes, taken out of the immunosuppressive environment. Hence, the question remained of whether monocytes would still respond to TLR8 agonist treatment while within this environment. To test this, we isolated white blood cells (WBCs) from CLL-patient peripheral blood and treated with or without TL8-506 for 48 h. We then measured surface expression of FcγR using flow cytometry and found significant upregulation of FcγRIIa and downregulation of FcγRIIb (Figure 3A), similar to that seen with isolated monocytes (Figure 2C). We also found significant increases in CD86, CD63 and CD11b, with no significant change in CD69 (Figure 3B), which indicate an increase in inflammatory activity in monocytes. CD86 is usually upregulated in activated cells and related to co-stimulatory signals for T cells [44], while CD63 is linked to activation, granule mobilization and potentially enhanced phagocytosis [45] and CD11b is an integrin enhanced by cell activation and linked to pro-inflammatory activity [46,47]. Hence, TLR8 agonist treatment can at least in part overcome the effects of a suppressive microenvironment.

### 2.4. TLR8 Agonists Modify the Phenotype of NLCs

Nurse-like cells (NLCs) support the survival and proliferation of CLL cells [14,15] and are seen as the CLL counterparts of tumor-associated macrophages [48]. To test whether TLR8 agonist treatment would have an effect on their tumor-supportive phenotype, we cultured CLL-patient WBCs for 14 days to derive NLCs [14,15], then treated the cultures for 48 h with or without TL8-506. Expression of CD31, CD163 and CD86 was measured in NLCs using flow cytometry.

**Figure 4:**
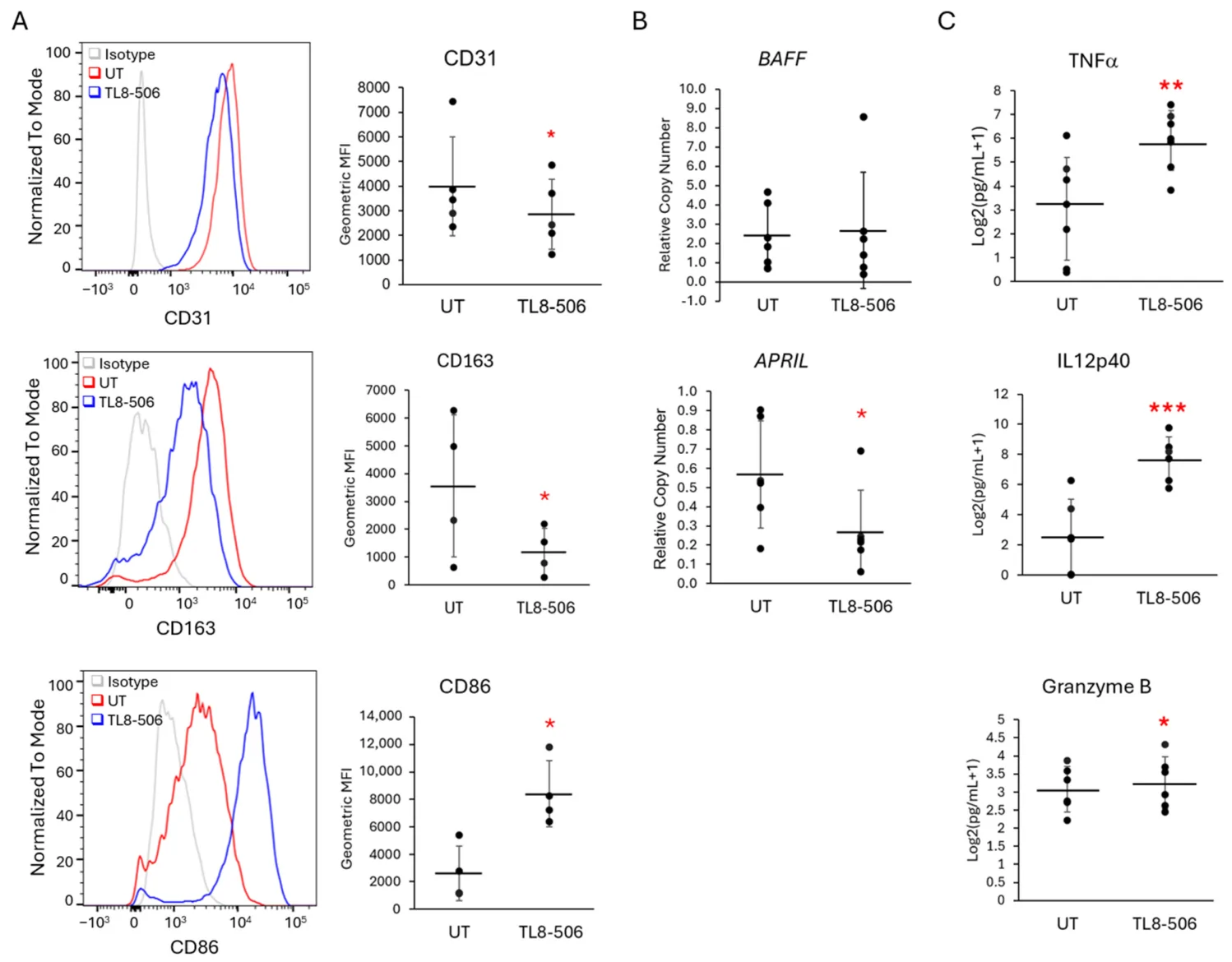
TLR8 agonists modify the phenotype of NLCs. WBCs were obtained from CLL patients and incubated for two weeks in collagen-coated plates to allow NLC development. Then, cultures were treated with or without 1 μM TL8-506 for 48 h. (**A**) Surface expression of *CD31*, *CD163* and *CD86* was measured by flow cytometry (n = 5 for *CD31*, n = 4 for *CD163*). (**B**) Expression of *BAFF* and *APRIL* was measured in NLCs by qPCR (n = 6). (**C**) Cleared supernatants were collected and TNFα, IL-12 (p40) and Granzyme B were measured using ELISAs. (n = 7 for TNFα, n = 6 for both Granzyme B and IL12p40). * *p* ≤ 0.05; ** *p* ≤ 0.01; *** *p* ≤ 0.001.

Results showed that surface levels of the checkpoint/adhesion molecule CD31 and the hemoglobin/haptoglobin receptor CD163, a standard marker for NLC phenotype and pro-tumoral activity [49], were significantly decreased (Figure 4A, top and middle panels, respectively). Conversely, the M1-related phenotypic marker CD86 was upregulated (Figure 4A, bottom panel). Because NLCs also provide pro-survival signaling (reviewed in [50]), we examined the expression of *BAFF* and *APRIL* using qPCR. Results showed no change in *BAFF* but a significant downregulation of *APRIL* (Figure 4B, upper and lower panels respectively). In addition, significantly increased levels of TNFα, IL12p40 and Granzyme B were found in supernatants of WBC-NLC cultures treated with TL8-506 (Figure 4C). Similar to monocytes, NLC results were qualitatively consistent for all patient samples, with the exception of one sample showing decreased Granzyme B after treatment. Hence, not only is TLR8 agonist treatment capable of restoring monocyte activation, it can also promote the activation of fully developed and differentiated NLCs.

### 2.5. TLR8 Agonist Treatment Enhances Activation and Antibody-Mediated Responses of NK Cells

**Figure 5:**
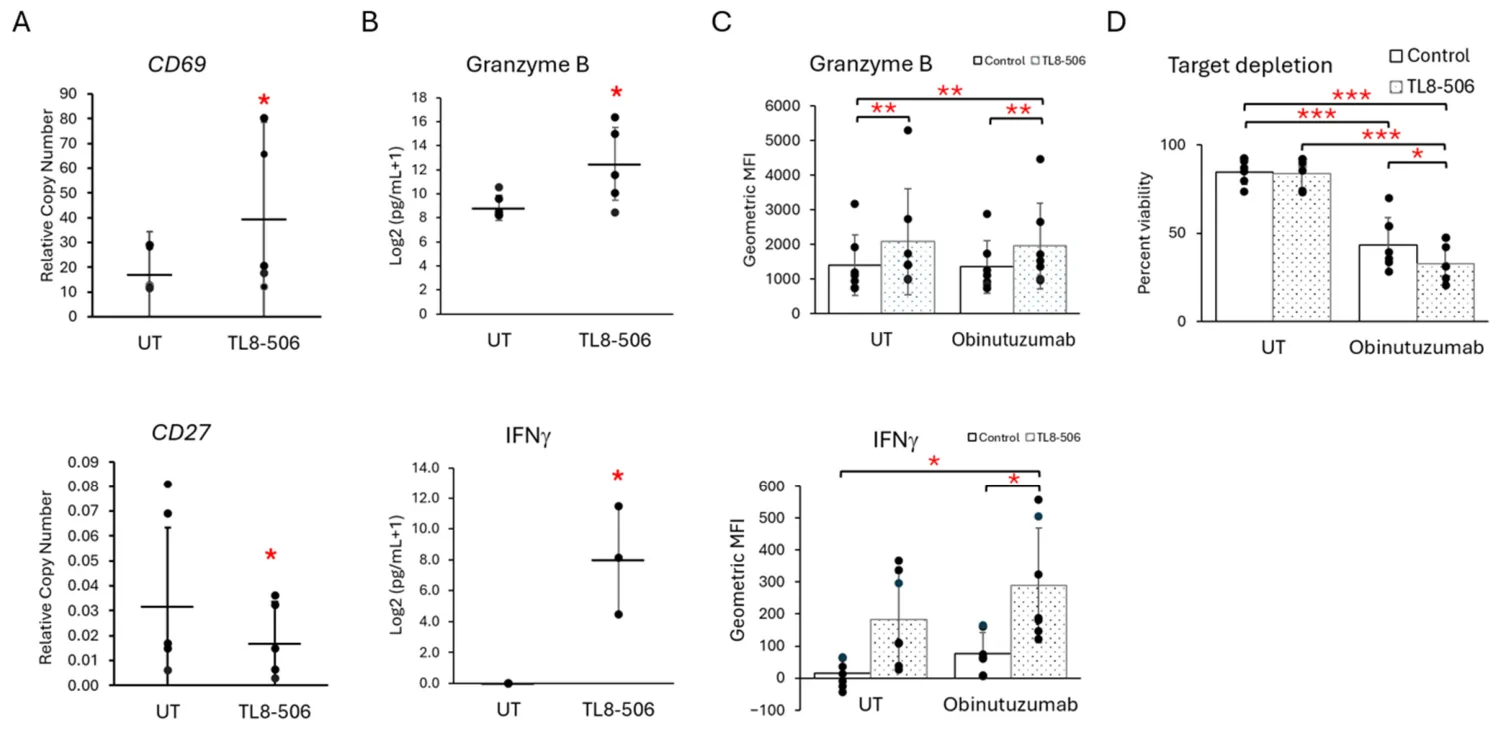
TLR8 agonist treatment enhances activation and antibody-mediated responses of NK cells. NK cells were isolated from CLL patients and incubated overnight with or without 1 μM TL8-506. (**A**) Expression of *CD27* and *CD69* was measured using qPCR (n = 7). (**B**) Following similar isolation and treatment, cleared supernatants were collected for measurement of Granzyme B (n = 6) and IFNγ (n = 3) by ELISA. (**C**) After overnight stimulation, NK cells were then co-cultured with obinutuzumab-opsonized or non-opsonized autologous B cells at 5:1 E:T ratio in u-bottom plates, and incubated in the presence of Brefeldin A for 4 h. Granzyme B and IFNγ were then measured in CD56^+^ cells using intracellular flow cytometry (n = 7). (**D**) NK cells were isolated and treated as above with or without TL8-506, then incubated with fluorescently labeled autologous B cells at a 5:1 E:T ratio in u-bottom plates. After 4 h, cytotoxicity was measured using flow cytometry (n = 6). * *p* ≤ 0.05; ** *p* ≤ 0.01; *** *p* ≤ 0.001.

As discussed above, CLL-patient NK cells have been found to show signs of suppression. Hence, we tested whether treatment with TLR8 agonist could help offset this. We isolated NK cells from CLL-patient blood samples, then incubated overnight with or without TL8-506. We then measured expression of the subset markers *CD11b* and *CD27* using qPCR. Results showed no change in *CD11b* (Supplementary Figure S3, left panel). However, there was a decrease in *CD27* (Figure 5A, top panel), suggesting a potential subset shift. Markers for activation (*CD69*) and degranulation (*CD107a*) were also tested using qPCR, with results showing a significant increase in *CD69* (Figure 5A, bottom panel). There was no change in CD107a (Supplementary Figure S3, right panel), although changes in surface expression were not measured.

Collectively, these results indicated a potential subset shift and greater activation. Hence, we next treated NK cells as above and measured IFNγ and Granzyme B in the supernatants. Results showed that TL8-506 consistently increased Granzyme B (Figure 5B, top panel). For IFNγ, of the seven samples analyzed, six showed detectable levels of the cytokine, while three of them showed an increase after TL8-506 treatment that were over the detection limit, even after dilution. The remaining three samples showed a significant increase in IFNγ upon TLR8 stimulation (Figure 5B, bottom panel). Next, we isolated and treated NK cells overnight as above, then incubated them for 4 h with autologous B/CLL cells that had been opsonized with obinutuzumab. Granzyme B and IFNγ were measured using intracellular flow cytometry.

Results showed that TL8-506 led to significant increases in Granzyme B (Figure 5C, top panel) and IFNγ (Figure 5C, bottom panel). However, we did not observe enhancement with TL8-506 in combination with the opsonized target cells. Next, to test the functional outcome of TLR8 agonist treatment, we measured the antibody-mediated cytotoxic function of NK cells. NK cells were isolated and treated as above, then incubated for 4 h with obinutuzumab-coated autologous B/CLL cells. Results showed that TL8-506 treatment modestly yet significantly enhanced NK-cell-mediated ADCC. Taken together, these results show that TLR8 stimulation promotes NK cell activation and function.

### 2.6. TLR8 Agonist Treatment Activates Neutrophils Without Increasing NET Formation

**Figure 6:**
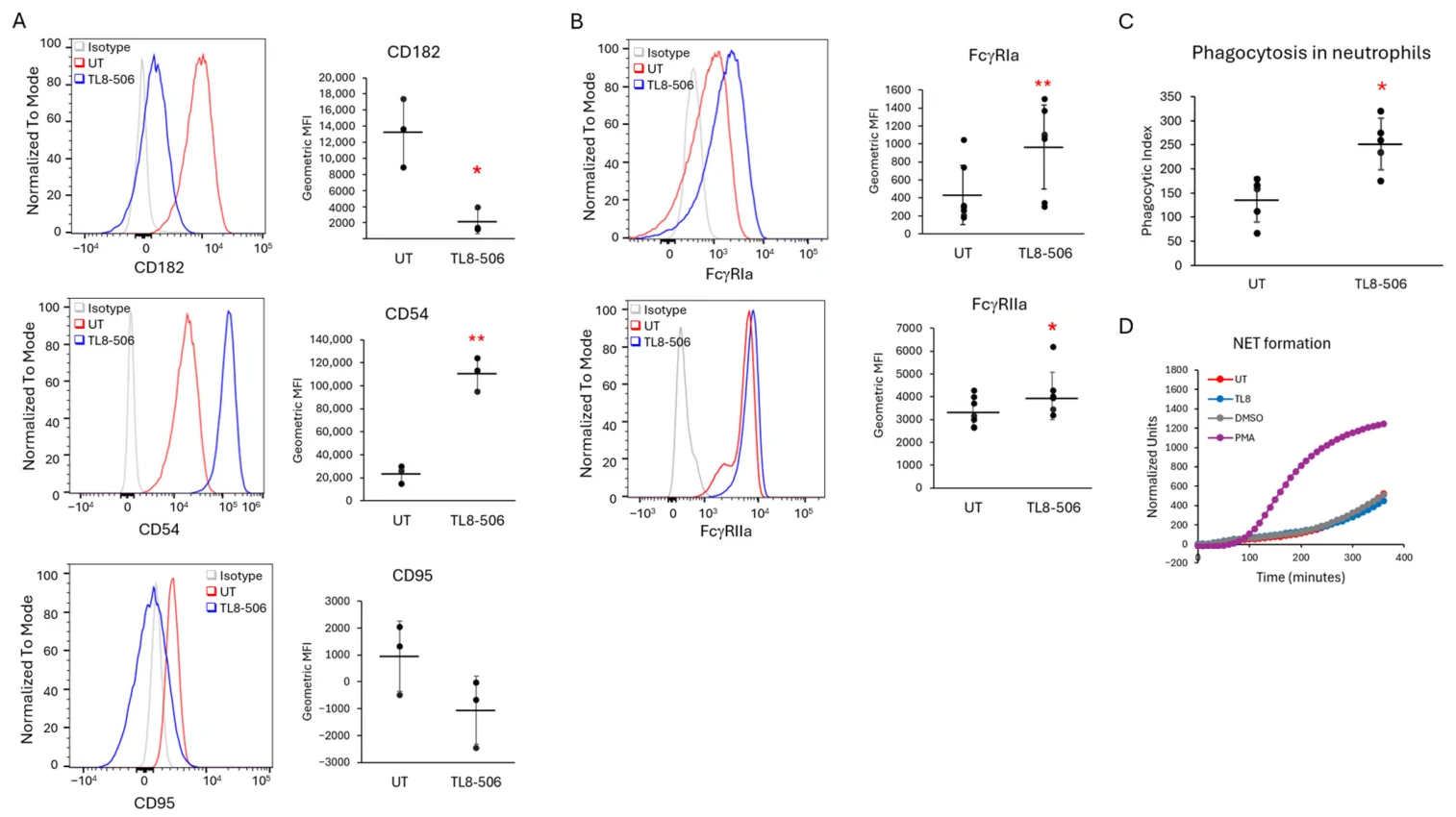
TLR8 agonist treatment activates neutrophils without increasing NET formation. WBCs from CLL patients were treated with or without 1 μM TL8-506. Expression of (**A**) CD182, CD54 and CD95 (n = 3) and (**B**) FcγRIa and FcγRIIa (n = 7) was measured by flow cytometry, at 24 or 48 h, respectively. (**C**) WBCs from CLL patients were treated with or without 1 μM TL8-506 overnight. Phagocytosis of opsonized sRBCs was evaluated at 4 h of co-culture, using CD66b staining to identify neutrophils (n = 5). (**D**) Enriched neutrophils from CLL patients were cultured in 96-well plates and treated with or without 1 μM TL8-506. NET formation was monitored every 10 min with Sytox Green for a total of 6 h using a plate reader. Phorbyl 12-myristate 13-acetate (PMA) treatment at 50 nM was used as a positive control. A representative curve of three individual experiments is shown. * *p* ≤ 0.05; ** *p* ≤ 0.01.

Because neutrophils have been shown to be aberrantly activated to possibly promote CLL disease [13,28,33], we tested whether TLR8 agonist treatment would worsen this or would help drive antitumor function. We began by incubating WBCs from CLL patients with or without TL8-506 for 24 h and measuring the neutrophil phenotypic markers CD182 and CD54 by flow cytometry. Results showed that levels of surface-expressed CD182 (N2-related marker) were significantly downregulated, while levels of the N1-related marker CD54 were upregulated (Figure 6A, top and middle panel, respectively). The Fas receptor (CD95) was also measured, showing a non-significant downregulation in our tests (Figure 6A, bottom panel).

We then measured FcγRIa and FcγRIIa and found that surface expression of each was significantly increased after 48 h of stimulation, as part of the assay done to measure the expression of FcγR in monocytes within WBCs (Figure 6B). Next, we examined FcγR-mediated function using phagocytosis assays. Results showed that TL8-506 treatment led to a significant enhancement of neutrophil phagocytic ability (Figure 6C). Finally, because NET formation may have pro-tumoral effects in CLL [33], we performed a kinetic assay to measure NET formation over time in enriched neutrophils. We treated freshly isolated neutrophils with or without TL8-506, comparing against treatment with the positive control phorbyl 12-myristate 13-acetate (PMA). Results showed that TL8-506 elicited no more NET/cell death formation than the untreated or DMSO-treated controls (Figure 6D). To further confirm this, we tested an additional two patient samples using confocal microscopy in conjunction with the Sytox assay. Results showed PMA-induced formation of cloud cells (an early stage of NET formation [51]) (Supplementary Figure S4). These results show that TLR8 agonist treatment can positively modulate neutrophil phenotype and activity without increasing NET formation.

## 3. Discussion

CLL has been widely known to produce a suppressive immune environment, hindering anti-leukemic activities of leukocytes. This is of particular relevance to monoclonal antibody therapy due to its heavy reliance on cellular responses (reviewed in [52,53]). Previously, we have observed that stimulation of TLR7/8 enhanced antibody-mediated responses of myeloid cells in healthy donors, leading to increased cytokine production and ADCC [37,54]. Due to CLL-related defects in myeloid cells [19], we first needed to ensure that CLL-patient monocytes and other innate immune cells were capable of responding to TLR8 agonists. Indeed, compared to responses found earlier with healthy-donor cells [37,54], patient monocyte responses were attenuated. However, the observed response helps confirm that patient monocytes are still capable of shifting away from their suppressed state with adequate stimuli. Overall, results showed that treatment with TLR8 agonist led to substantial activation of monocytes, NK cells and neutrophils, largely offsetting their suppressed phenotypes. This activation and phenotype shift was accompanied by modulation of FcγR expression and activity.

The use of innate response activators as a therapeutic approach has been explored since the last century [55], with the goal of transforming the microenvironment and promoting anti-tumoral responses. Indeed, rudimentary forms were tested in the late 1800s [56]. Within the past several decades, TLR agonists have been studied as an alternative therapeutic option for CLL. For example, TLR7 agonist treatment was shown to induce apoptosis in CLL cells [57]. However, the effects of TLR8 activation, especially on cells within the CLL microenvironment, have not been fully explored.

The specific mechanisms by which TLR8 agonists may overcome suppression of CLL-associated immune effectors will require further exploration. However, one possibility is that of downregulating the inhibitory/checkpoint receptor CD31. This downregulation would be highly pertinent to CLL, perhaps not only reducing negative signaling on effector cells but also reducing CD38 signaling in CLL cells; CD31 has been shown to help support CLL survival through linkage with CD38 [58]. It should also be remembered that CD31 performs a variety of activities within multiple cell types [59], while our study measured it only in CD14^+^ cells. Within the context of antibody therapy, we have previously shown that CD31 acts as a checkpoint molecule and negatively affects antibody-mediated phagocytosis [39]. Our current results showed only a trend toward *CD31* transcriptional downregulation, but a significant reduction in surface expression. The significant reductions in the checkpoint receptor [60] FcγRIIb and in the negative regulator SHIP-1 may also have contributed. Interestingly, although *CD200R* showed significant downregulation with qPCR, surface expression remained unchanged. Perhaps this represents a temporal difference or a reduced turnover and increased recycling of the protein. This phenomenon could be the subject of future experiments.

In addition to the effects on negative regulatory molecules, we saw an increase in the expression of the activating receptor, FcγRIIa, which further indicates a shift toward enhanced antibody-mediated responses. Indeed, TLR8 stimulation in monocytes increased the production of TNFα after FcγR clustering, suggesting an additive effect between the two treatments.

Due to their importance in CLL, the effects on developed NLCs were assessed. Similar to what we observed in monocytes, CD31 expression was downregulated after TL8-506 treatment. In addition, transcriptional expression of *APRIL*, highly linked to CLL disease progression [61], was reduced. Furthermore, cytokines and Granzyme B were released, suggesting a potential cytotoxic phenotype in NLCs induced by the agonist. As such, results suggest that TLR8 agonist treatment can modulate the phenotype and activity of NLCs, skewing them away from a tumor-supportive phenotype. Previously we have shown similar results with IFNγ treatment [62], and results from the present study further support the idea that NLCs are malleable.

NK cells are also highly relevant to therapeutic anti-CD20 antibody responses [63]. However, CD27 has been reported to be increased in CLL-patient NK cells [23], suggesting an immature phenotype. Our present study showed that TLR8 stimulation led to decreased transcript levels of *CD27* as well as to an increase in *CD69*, an early activation marker [64]. NK cells appear capable of directly responding to TL8-506, as shown by enhanced release of IFNγ and Granzyme B. In addition, there is improved ADCC against obinutuzumab-coated autologous B/CLL cells. Our results are in concordance with previous reports testing VTX-2337 and other TLR8 agonists against healthy-donor NK cells [65,66].

Neutrophils have also been recognized as an active part of the tumor microenvironment in CLL, so it was important to examine their responses to TLR8 agonist treatment. Upon TLR8 stimulation, both CD54 and FcγRIa were increased. Interestingly, CLL-patient neutrophils have been reported to have higher levels of both receptors compared to healthy donors [13]. CD182/CXCR2, the IL-8 high affinity receptor, has been shown to be induced by CLL-patient-derived plasma in healthy-donor neutrophils [33] and has been linked to the production of NETs [67]. Of note, the increase in both CD54 and CD64 we observed upon TLR8 stimulation was paired with a reduction in CD182 expression, which is indicative of a change in neutrophil phenotype.

It has previously been shown that healthy-donor neutrophils are capable of performing phagocytosis of obinutuzumab-coated B-cell targets [63]. Here, we found that neutrophils from CLL patients upon treatment with TL8-506 showed upregulation of FcγRIa and FcγRIIa. In addition, their ability to phagocytose antibody-coated targets was increased. Importantly, we did not see increased NET formation, which may have favorable implications for the use of TLR8 agonists against CLL. NETs may serve to promote CD5^+^ B-cell proliferation (reviewed in [68]), and it has been reported that neutrophils from CLL patients show greater NET formation than healthy-donor counterparts upon stimulation [33]. Given this, an agonist that leads to neutrophil activation without increasing NETs would be preferred. In addition, NET formation can drive cytokine production (reviewed in [69]), which might add to the already-higher cytokine levels seen in CLL patients [70]. This may increase the risk of inducing cytokine release syndrome. Alternatively, some studies suggest that NET formation can drive M2 polarization in hypoxic environments and in some cancers (reviewed in [71,72]). Because nurse-like cells already exhibit an M2-like phenotype [14,15,16,73], an additional M2-promoting stimulus via NETs may counteract the M1-promoting effect of TLR8 agonist treatment. Although we saw no agonist-driven NET formation in the current study, further exploration of the role of NETs within CLL would be informative, especially within a model in vivo.

One concern with antibody therapy for CLL is cytokine release syndrome (CRS) [74,75,76], which would likely be exacerbated by the co-administration of a TLR8 agonist. However, it may be possible to reduce the likelihood of CRS by staggering the administration of agonist and antibody, or by using one or more additional strategies. For example, Aichhorn et al. [77] developed a masked version of a TLR7/8 agonist that becomes activated in acidified endosomes. This could be targeted to myeloid-lineage cells via DNA origami [78] or other targeting systems (reviewed in [79,80]). Specificity could be further enhanced via linkage to an antibody that targets CD14 or other myeloid-enriched surface proteins. This targeting would also help bypass any potential pro-tumoral direct effects of TLR8 agonists on CLL cells. For example, treatment of CLL cells with the TLR7/8 agonist R848 has been shown to induce short-term proliferation [81].

There are several limitations to this study that should be considered for future experiments. Firstly, the possibility exists that the TLR8 agonist we used, TL8-506, may not be fully specific for TLR8. Given this, it can be stated that it is one of the most selective TLR8 agonists to date. For example, the CU-CPT9a inhibitor of TLR8 was tested against TL8-506 in PBMCs, with the inhibitor abrogating IL-6 production [82]. The CU-CPT9a inhibitor had previously been verified as highly selective for TLR8 compared to TLRs 2, 4, 5, 7 and 9 [83]. From a functional perspective regarding specificity between TLR8 and the related TLR7, TLR7 also employs MyD88 [84] but it was found by Vierbuchen et al. [85] that activation of TLR8 but not TLR7 could drive inflammasome signaling and subsequent IL-1β release. In our results (Figure 1C), we found strong production of IL-1β.

Going hand-in-hand with this, the agonist may affect the CLL-patient cell types in differential manners, especially with regard to viability or activation. This itself may contribute to the observed results and could be studied in detail in future experiments. In addition, we were not able to stain for and measure every cell type. For example, we did not account for NKT cells, which, although low in proportion [86], may have played a role. From a larger perspective, the patient-to-patient variability itself may greatly affect the efficacy of TLR8 agonist treatment. To definitively generalize across all CLL patients, we would need to obtain and test sample sizes sufficiently large to permit statistical testing among and between the disease variants. This extends beyond our study, where we obtained de-identified CLL-patient samples and tracked CLL cells via CD5^+^/CD19^+^. As such, generalization would need to be done with caution and further testing.

Another limitation of this study is that it examined samples from patients who were previously untreated and who had relatively low counts of circulating tumor cells. As such, results may not be fully applicable to later-stage patients or patients who had previously undergone treatment. In addition, we used the glycoengineered anti-CD20 antibody obinutuzumab [87] and did not examine other antibodies such as rituximab. More generally, our experiments were done using patient samples in an ex vivo setting. Although well-characterized and standardized [14,15], this culturing and testing system may not fully represent the responses that would be seen within the patients, especially with regard to potential resistance or relapse. Hence, future experiments should include the testing of TLR8 agonists in whole-organism models of CLL. This may include mouse models [88] or perhaps canine clinical trials, as dogs are known to spontaneously develop CLL [89,90,91,92]. Here, not only might diseased pets benefit, but the results may be more relevant to humans [93]. Targeted and masked iterations of the TLR8 agonist could also be examined.

## 4. Materials and Methods

### 4.1. PBMC and WBC Isolation

De-identified whole-blood CLL patient samples were provided by the Hematology Tissue Bank of The Ohio State University Comprehensive Cancer Center under IRB-approved protocol 1997C0194 and in accordance with the Declaration of Helsinki. To isolate white blood cells (WBCs), blood samples were incubated with HetaSep (Stemcell Technologies, Cambridge, MA, USA) in a 1:5 ratio for 30 min at 37 °C, as per manufacturer instructions. Then, the WBC layer was collected and remaining red blood cells were lysed with ammonium-chloride-potassium (ACK) buffer before washing and counting using the Cellometer Auto 2000 (Nexcelom Biosciences, Lawrence, MA, USA). Cells were resuspended in RPMI (Gibco, ThermoFisher Scientific, Waltham, MA, USA) supplemented with 10% FBS (VWR, Radnor, PA, USA), 2 mM L-glutamine (Gibco, ThermoFisher Scientific), and 100 U/mL Penicillin-Streptomycin (Gibco, ThermoFisher Scientific) at 10 × 10^6^ cells/mL. For activation, cells were incubated in polystyrene tubes (Falcon, Corning, NY, USA) and treated with TL8-506 (InvivoGen, San Diego, CA, USA), at a final concentration of 1 μM for the indicated time-points.

For enriched monocytes, B cells and NK cells, magnetic isolation was used, using manufacturer’s instructions [94,95,96]. Briefly, peripheral blood mononuclear cells (PBMCs) were isolated using lymphocyte separation medium (Corning, Corning, NY, USA) to create a density gradient via centrifugation. The PBMC layer was removed and washed with RPMI. PBMCs were incubated with magnetic anti-CD14 or anti-CD19 selection beads (Miltenyi Biotec, Waltham, MA, USA) for 15 min on ice. Following this, cells were centrifuged and resuspended in MACS buffer (Miltenyi Biotec) and added to an LS selection column. After three washes, cells were recovered from the column, washed with media and counted. Monocytes were passed through a second column to increase enrichment; they were used at a concentration of 1 × 10^6^/mL in complete RPMI media, while B cells were resuspended at 5 × 10^6^/mL for ADCC assays.

For NK cells, the EasySep^TM^ Human CD56 isolation kit II was used (Stemcell Technologies) per manufacturer’s instructions. PBMCs were resuspended in MACS buffer and incubated with the antibody cocktail followed by an incubation with rapidspheres at room temperature. Then, MACS buffer was added and the cells incubated in the magnet to allow separation. Remaining cells in suspension were then discarded, and the isolated cells were washed three more times with MACS buffer. Resulting cells were then counted and resuspended in complete RPMI media.

### 4.2. NLC Cultures

For nurse-like cell formation, WBCs from CLL patients were incubated at 10 million/mL in complete RPMI using collagen-coated plates (Gibco) for two weeks, supplying fresh media as needed, as described previously [14,95]. After incubation, the culture was treated for 48 h with TL8-506 1 μM. For flow cytometry, cells were washed and NLCs detached using collagenase at 0.1% in HBSS with no calcium or magnesium, supplemented with 10 μg/mL of Polymyxin B (Calbiochem, Millipore Sigma, Burlington, MA, USA) for 1 h at 37 °C. Cells then were incubated with Fc block and stained for flow cytometry. In addition to extracellular staining, intracellular staining for detection of CD68 was done using the BD cytofix/cytoperm kit (BD Biosciences, San Jose, CA, USA) per manufacturer instructions. For gene expression, WBC culture plates were thoroughly washed after TL8-506 treatment with PBS. Plates were constantly monitored during washes to visually check presence of non-adherent cells/lymphocytes. RL buffer was added directly to the plate and further proceeded to RNA isolation.

### 4.3. Quantitative Real-Time PCR

To determine the expression at transcriptional levels after TLR8 stimulation, treated cells were pelleted and lysed with RL buffer. For RNA isolation, the total RNA isolation kit (Norgen Biotek Corp, Thorold, ON, Canada), following the manufacturer instructions. After RNA isolation and quantification, cDNA was obtained using the High-Capacity cDNA Reverse transcription kit (Applied Biosystems, Thermo Fisher Scientific, Waltham, MA, USA). Gene expression was then evaluated by real-time PCR using SYBR Green (Applied Biosystems, ThermoFisher Scientific) in a QuantStudio 3 PCR machine (Applied Biosystems). GAPDH was used as housekeeping control, and relative copy number calculated as: RCN = (2^(−∆Ct)^) × 100 [97]. Measurements were done in duplicate. The primers used are listed in Supplementary Table S1.

### 4.4. Western Blot

Stimulated monocytes were centrifuged and lysed with TN1 lysis buffer as previously described [39]. Protein was quantified using the *DC* Protein Assay according to manufacturer’s instructions (Bio-Rad, Hercules, CA, USA) and boiled in 5x SDS sample buffer (150 mM Tris; 11.5% SDS; 0.05% bromophenol blue; 50% glycerol; 1% 2-mercaptoethanol). For analysis, electrophoresis was done in SDS-PAGE and then proteins were transferred to a nitrocellulose membrane using the Trans-Blot Turbo Transfer System (Bio-Rad). Membranes were blocked with the Li-COR Blocking solution (Li-COR Biotechnology, NE, USA) and incubated with primary antibodies overnight at 4 °C in Li-COR antibody diluent. The following primary antibodies were utilized: rabbit anti-human FcγRIa (clone EPR4623, Abcam/Epitomics, Waltham, MA, USA); rabbit anti-human FcγRIIa (product T3562, Abcam/Epitomics), rabbit anti-human CD32b (clone E4D2F, Cell Signaling Technologies, Danvers, MA, USA), rabbit anti-human FcγRIIIa (clone EPR4333, Abcam/Epitomics); rabbit anti-human FcεRIγ1 (product 06-727, Upstate, Sigma-Aldrich, Millipore Sigma, Burlington, MA, USA); mouse anti-human GAPDH (Clone D4C6R, Cell Signaling Technology); and mouse anti-human calreticulin (Clone FMC 75, Enzo Life Sciences, Farmingdale, NY, USA). Membranes were then washed and incubated with fluorescently conjugated secondary antibody (IRDye 800CW goat anti-rabbit, or IRDye 680RD goat anti-mouse antibodies; Li-COR) for 1 h at room temperature. Finally, membranes were developed using the Odyssey CLx (Li-Cor). For densitometry analysis, ImageJ 1.53t was used.

### 4.5. Enzyme Linked Immunosorbent Assay (ELISA)

Cultured cells, treated with TL8-506 at the specified times, were centrifuged and cleared supernatants were collected and stored at −80 °C prior to use. For antibody-mediated cytokine TNFα release in monocytes, whole human IgG (Jackson ImmunoResearch Labs, West Grove, PA, USA) was heated up at 63 °C for 90 min, then kept on ice before use. Enriched monocytes were treated with TL8-506 1 μM for 24 h and then heat-aggregated IgG (ΔIgG) was added at a concentration of 350 μg/mL; cells were incubated for further 24 h before collection of the supernatants.

DuoSet ELISA kits for Human TNFα, IL12p40, IFNγ, IL1-β, and Granzyme B (R&D Systems, Minneapolis, MN, USA) were used according to manufacturer’s instructions. Dilutions of supernatants were done with reagent diluent as needed, and samples were plated in duplicate. For acquisition, absorbance was measured with the Synergy HTX plate reader (Biotek, Winooski, VT, USA). Calculations were done using the appropriate standard curve with a four-parameter adjustment. Due to the variability of cytokine release, data are presented as Log_2_ (pg/mL). Samples outside the detection range, even after dilutions, were not included in the analysis.

### 4.6. Flow Cytometry

After cell stimulation, cells were collected and washed with PBS. Then, cells were transferred into v-bottom plates and incubated with 10 μg/mL whole human IgG in PBS 15 min on ice. Then, the appropriate antibody cocktail (Supplementary Table S2) was added, and cells incubated for 30 min. Cells were then washed and fixed with 2% paraformaldehyde in staining buffer (1% BSA in PBS) and stored at 4 °C until acquisition. Samples were acquired using the LSR-Fortessa (BD Biosciences) or the Cytek Aurora 5-laser analyzer (Cytek, Fremont, CA, USA), at the Flow Cytometry Shared Resource at The Ohio State University Comprehensive Cancer Center. Results were further analyzed using FlowJo version 10.10.1 (Ashland, OR, USA). Complexity index values for the panels are included in Supplementary Table S2. Gating strategies are shown in Supplementary Figures S5 (isolated monocytes), S6 (monocytes and neutrophils in WBC cultures), S7 (NLCs in WBC cultures), S8 (NK-cells and target cells) and S9 (neutrophils in WBC cultures). Analyses of results were done using isotype controls to subtract background fluorescence, when calculating Geometric Mean Fluorescence Intensities (MFIs). In general, all analyses included the elimination of doublets and dead cells. However, for ADCC, dead cells were included as part of the measurements. In addition, doublets were not removed from NLC analysis because these cells have been shown to have a high size and complexity.

### 4.7. Antibody-Dependent Cellular Cytotoxicity

For antibody-dependent cellular cytotoxicity (ADCC), isolated NK cells from CLL patients were incubated with TL8-506 at 1 μM overnight. Then, isolated B cells from the same patients were stained for 10 min with Cell Trace Far Red (Invitrogen, ThermoFisher Scientific, Waltham, MA, USA) and washed; cells were opsonized with obinutuzumab at 10 μg/mL (Selleck Chemicals, Houston, TX, USA) on ice for 30 min. Cells were centrifuged and media replaced, then counted and incubated in u-bottom 96-well plates in an effector to target ratio (E:T) of 5:1 in 100 μL of phenol-free, complete RPMI. Cells were then lightly centrifuged and incubated for 4 h at 37 °C.

For measurement of survival through flow cytometry, cells were centrifuged and resuspended in Annexin V binding buffer, stained with Annexin V—FITC (BD Biosciences) for 15 min, and then diluted with binding buffer. Sytox blue (Invitrogen, ThermoFisher Scientific) was added to the tubes before acquisition. Due to low numbers of NK cells after isolation, duplicates were done for only two out of the six patient samples.

### 4.8. Phagocytosis

To measure phagocytic activity in neutrophils, the protocol was based on Karsten et al. [98]. As targets, sheep red blood cells (sRBCs) were stained with PKH-26 (Sigma-Aldrich, Millipore Sigma) and opsonized with rabbit anti-sheep antibody, as previously described [62]. sRBCs were used within two weeks of preparation and thoroughly washed before use.

For the assay, WBCs from CLL patients were incubated overnight with 1 μM TL8-506. This shorter incubation was done to reduce potential noise from spontaneous cell death in neutrophils, and then cells were mixed with 1 μL of packeted fluorescently labeled, opsonized sRBCs. Cells were spun down and incubated for 4 h. After incubation, free and non-internalized sRBCs were lysed with ACK buffer and washed. Cells were then Fc-blocked with human IgG for 15 min on ice before staining with CD66b—FITC antibody (clone QA17A51, Biolegend, Revvity, San Francisco, CA, USA). After 30 min, cells were washed and fixed with 2% paraformaldehyde. To determine the phagocytic activity, the number of engulfed sRBCs was counted in 100 CD66b-positive cells using an Olympus DP71 fluorescence microscope (Olympus, State College, PA, USA). The counts were done independently by two or three different individuals in a blinded fashion. Phagocytosis of non-opsonized, fluorescent sRBCs was subtracted to account for non-antibody-mediated phagocytosis.

### 4.9. NET Formation Assay

To evaluate neutrophil extracellular traps (NETs) a kinetic assay was done according to a previously established protocol [99,100]. For this, neutrophils were isolated directly from freshly drawn blood using the neutrophil isolation kit (Stemcell Technologies) according to the manufacturer’s instructions. Here, however, the amount of antibody used per mL was increased from 50 to 75 μL to reduce B cell retention in the isolated cells (personal communication with Dr. Fabienne Lucas). Then, isolated cells were counted and seeded at 100,000 cells/100 μL of HBSS plus calcium and magnesium (Gibco). As controls, DMSO or PMA at 50 nM was added, as well as 1 μM TL8-506. For measuring activity, Sytox Green (Invitrogen, ThermoFisher Scientific) was added at a concentration of 0.5 μM final concentration. Fluorescent emission was measured in triplicate every 10 min while the plate was maintained at 37 °C using the Synergy HTX plate reader at an excitation of 485 nm, and emission set at 528 nm. Time 0, non-stimulated or DMSO control values were subtracted as baseline and subtracted from measurements as normalization.

As controls, for two patients, confocal microscopy images with staining for dsDNA and for myeloperoxidase [30,101] were acquired in conjunction with the Sytox Green measurements (Supplementary Figure S4). For this, neutrophils were seeded at 150,000 cells per well in HBSS plus calcium and magnesium (Gibco), using 8-well imaging culture slides (Ibidi, Gräfelfing, Germany). Cells were then treated with 50 nM PMA, 1 μM TL8-506 or vehicle (DMSO) for 3 h to allow NET formation, followed by fixation. For staining, slides were blocked with goat serum before adding mouse anti-dsDNA (clone 35I9 DNA, Abcam) and rabbit anti-MPO (clone EPR20257, Abcam) at 1:100 dilutions. Slides were incubated overnight, then washed and incubated for an hour with Alexa Flour 488 goat anti-rabbit (Ref A11008, Invitrogen, ThermoFisher Scientific), Alexa Flour Plus 555 goat anti-mouse F(ab’)2 (ref A48287, Invitrogen, ThermoFisher Scientific), and Alexa Flour 647 Wheat Germ Agglutinin (WGA, for plasma membrane staining: Ref W32466, Invitrogen, ThermoFisher Scientific) for 1 h. Slides were then washed and kept in PBS until acquisition. Images were acquired using a Nikon AXR confocal microscope at the Microscopy shared resource at The Ohio State University. Images were taken as z-stacks and then analyzed using ImageJ 1.53t. For the analysis, z-stacks were prepared by eliminating images with glass background, and slightly adjusting for brightness; then, z-stacks were merged to obtain a flat image and scale bars were added.

### 4.10. Statistical Analysis

For primary samples treated with vehicle versus TLR8 agonist, paired one-tailed t-tests were done [102] using Microsoft Excel, as prediction of differences prior to each experiment was done, based on our earlier work with TLR7/8 and TLR8 agonists [37,38,54]. For normality, Shapiro testing, as well as normality and QQ plots were done in R Studio version 2025.05.0+496. Linearity of the QQ plot (expressed as R^2^) was obtained using Microsoft Excel. Results of normality testing, *p*-values, mean/medians, confidence intervals, as well as predicted alternative hypotheses are included in Supplementary Table S3. For Figure 2D and Figure 5C,D, assessment of normality and mixed effects models, accounting for the sample from the same subject undergoing various treatments, and the Hochberg method to adjust for multiple testing, were done using SAS software 9.4 (SAS Institute, Cary, NC, USA). General statistical summaries of patient demographics have been included in Supplementary Table S4.

## 5. Conclusions

Results from the present study show that the TLR8 agonist TL8-506 has the potential to alter the phenotypes and activities of innate immune effector cells within the context of CLL. In particular, FcγR-mediated responses appear stronger after agonist treatment. Hence, TLR8 agonist treatment may be of benefit as an adjuvant treatment for antibody therapy, and further exploration may ultimately be of clinical benefit to CLL patients.

## Data Availability

Dataset available on request from the authors.

## Supplementary Materials

The following supporting information can be downloaded at https://www.mdpi.com/article/10.3390/ijms27188003/s1.

## Abbreviations

The following abbreviations are used in this manuscript:

ADCC	Antibody-dependent cellular cytotoxicity
ADCP	Antibody-dependent cellular phagocytosis
APRIL	A proliferation-inducing ligand
BAFF	B-cell activating factor
CD*nn*	Cluster of Differentiation *nn*
CLL	Chronic lymphocytic leukemia
DLBCL	Diffuse large B-cell lymphoma
ELISA	Enzyme-linked immunosorbent assay
FBS	Fetal bovine serum
FcγR	Fc gamma receptor
IFN	Interferon
IRB	Institutional Review Board
MACS	Magnetic-activated cell sorting
NK	Natural killer
NLC	Nurse-like cell
PCR	Polymerase chain reaction
RPMI	Roswell Park Memorial Institute
SDF	Stromal-derived factor
SIRPα	Signal regulatory protein alpha
sRBC	Sheep red blood cell
TLR	Toll-like receptors
TNFα	Tumor necrosis factor alpha
WBC	White blood cell
