# Supplementary material for: TLR8 Stimulation Leads to Microenvironment Activation and Enhanced Antibody-Dependent Cellular Responses in CLL
Source: Int J Mol Sci. 2026 Sep 9;27(18):8003. doi: 10.3390/ijms27188003 (PMC13606378; doi:10.3390/ijms27188003)
Supplement: Supplementary file 1 [file ijms-27-08003-s001.zip › ijms-4442949-supplementary.pdf]

| Target | Forward (5’->3’) | Reverse (5’->3’) |
|---|---|---|
| *GAPDH* | ACTTTGGTATCGTGGAAGGACT | GTAGAGGCAGGGATGATGTTCT |
| *FcγRIa* | GGCAAGTGGACACCACAAAGGCA | GCTGGGGGTCGAGGTCTGAGT |
| *FcγRIIa* | TTGCTGCTGCTGGCTTCTGC | GTAGCTGGGCTGCGTGTGGG |
| *FcγRIIb* | AGCGGATTTCAGCCAATCCC | CATCCGGGTGCATGAGAAGT |
| *FcγIIIa* | CCTCTCCACCCTCAGTGACCCG | TGGAGCAACAGCCAGCCGAT |
| *FcεR1γ* | CAAGCAGCGGCCCTGGGAG | TTCCTGGTGCTCAGGCCC |
| *CD31* (*PECAM1*) | ATTGCAGTGGTTATCATCGGAGTG | CTCGTTGTTGGAGTTCAGAAGTGG |
| *SIRPα* | TGCCAGAGAAATAACACAGGACA | CTGGCATACTCCGTGTGGCT |
| *CD200R* | GAGCAATGGCACAGTGACTGTT | GTGGCAGGTCACGGTAGACA |
| *SHIP1* | TCCAGCAGTCTTCCTCACCT | GCTTGGACACCATGTTGATG |
| *SHIP2* | GGGACATTTGAGGTTGGAGTTAC | GCACTTTGAGGAAGTTGATGTT |
| *CD11b* | GAGTCCAACGCTAATGTCAAGG | CCCGTAGAGAACAGCATCACAC |
| *CD69* | GGTCCTTCCAAGTTCCTGTCC | TTTCCTCTGGTAGCCAACCCA |
| *CD27* | TGTCGGCACTGTAACTCTGGT | GTTGCGAACGAGAAGACCTGG |
| *CD107a* | ACCTTTTCCCCAATGCGAGC | TCACAGCGTGTCTCTCCCC |
| *APRIL* | CTGCACCTGGTTCCCATTAAC | AAGAGCTGGTTGCCACATGA |
| *BAFF* | ACCGCGGGACTGAAAATCT | CACGCTTATTTCTGCTGTTCTGA |

**Supplementary Table S1.** Primers used for real-time PCR.

| | | |
|---|---|---|
| Panel: Fcy Receptors in monocytes<br><br>Complexity index: 1.69 (LSR Fortessa – 5 laser) | | |
| Target - Fluorophore | Clone | Supplier |
| CD14 – Alexa Flour 647 | HCD14 | Biolegend |
| CD64 – PerCP Cy5.5 | 10.1 | Stemcell |
| CD32a – FITC | IV.3 | Biolegend |
| CD32b – PeCy7 | S18005H | Biolegend |
| CD16 – Brilliant Violet 785 | 3G8 | Biolegend |
| Live dead Blue | | Invitrogen |
| Panel: Regulatory receptors molecules in monocytes<br><br>Complexity index: 1.70 (LSR Fortessa – 5 laser) | | |
| Target - Fluorophore | Clone | Supplier |
| CD14 – FITC | HCD14 | Biolegend |
| CD31 – PeCy7 | WM59 | Biolegend |
| CD200R – PE | OX108 | eBiosciences (ThermoFisher) |
| SIRP-α (CD172a) – AF647 | SE5A5 | BD Biosciences |
| HLA-DR PerCP | L243 | Biolegend |
| Live dead Blue | | Invitrogen |
| Panel: NK cells cytokine production<br><br>Complexity Index: 1.09 (LSR Fortessa – 5 laser) | | |
| Target - Flurophore | Clone | Supplier |
| CD56 – BV605 | HCD56 | Biolegend |
| CD107a – PeDazzle 594 | H4A3 | Biolegend |
| Granzyme B – AF700 | GB11 | BD Biosciences |
| IFNγ – PerCP | 4S.B3 | Biolegend |
| Live Dead Blue | | Invitrogen |
| Panel: WBCs Fcγ Receptors | | |

| Complexity Index: 1.79 (LSR Fortessa – 5 laser) | | |
|---|---|---|
| Target - Fluorophore | Clone | Supplier |
| CD11b – BV510 | M1/70 | Biolegend |
| CD14 – AF647 | HCD14 | Biolegend |
| CD15 – PE | HI98 | Biolegend |
| CD16 – BV711 | 3G8 | Biolegend |
| CD19 – BV421 | HIB19 | Biolegend |
| CD32a - FITC | IV.4 | Stemcell |
| CD32b – PeCy7 | S18005H | Biolegend |
| CD64 – PerCPCy5.5 | 10.1 | Biolegend |
| Live Dead Blue | | Invitrogen |
| Panel: WBCs cell activation<br>Complexity Index: 1.73 (LSR Fortessa – 5 laser) | | |
| CD14 – BV421 | HCD14 | Biolegend |
| CD 15 – AF488 | MC480 | Biolegend |
| CD11b – AF700 | M1/70 | Biolegend |
| CD16 – BV711 | 3G8 | Biolegend |
| CD19 – PeCy7 | HIB19 | Biolegend |
| CD56 – BV605 | HCD56 | Biolegend |
| CD63 – PE | H5C6 | Biolegend |
| CD69 – BV785 | FN50 | Biolegend |
| CD86 – APC | IT2.2 | Biolegend |
| Live Dead Blue | | Invitrogen |
| Panel: neutrophil activation<br>Complexity index: 2.08 (5-laser Cytek Aurora) | | |
| Target - Fluorophore | Clone | Supplier |

| | | |
|---|---|---|
| CD15 – Pacific Blue | W6D3 | Biolegend |
| CD66 – PerCPCy5.5 | QA17A51 | Biolegend |
| CD95 SparkBlue 574 | DX2 | Biolegend |
| CD182 – FITC | 5E8/CXCR2 | Biolegend |
| CD54 – PE | HCD54 | Biolegend |
| CD11b – Brilliant Violet 510 | M1/70 | Biolegend |
| CD16 – Brilliant Violet 711 | 3G8 | Biolegend |
| CD45 – APC | HI30 | BD Pharmingen (Waters Bio) |
| CD14 – PeCy7 | M5E2 | BD Pharmingen (Waters Bio) |
| Live dead Blue | | Invitrogen |

**Supplementary Table S2.** Antibody panels used for flow cytometry.

Part A: Paired, two-group comparison statistical data. Normality was evaluated by the Shapiro-Wilk test . Paired, non normal Wilcoxon testing was done for samples not showing normality.

| Figure | Measure | Marker | Sample size | Shapiro-Wilk p-value | QQplot $R^2$ | t-test p-value | Wilcoxon test p-value | Mean/Median | 95% Confidence Interval | | Alternative hypothesis |
|---|---|---|---|---|---|---|---|---|---|---|---|
| | | | | | | | | | Lower | Upper | |
| 1.a | qPCR | CD31 | 12 | 0.411 | 0.971 | 0.0982 | | 11.20 | -1.32 | 10.21 | UT>TL8 |
| 1.a | qPCR | CD200R | 10 | 0.001 | 0.637 | | **0.0010** | 2.99 | 2.64 | Inf | UT>TL8 |
| 1.a | qPCR | SHIP1 | 11 | 0.369 | 0.959 | **0.0022** | | 2.99 | 1.51 | 4.48 | UT>TL8 |
| 1.a | qPCR | SIRPα | 12 | 0.497 | 0.837 | 0.0009 | | -5.11 | -7.37 | -2.86 | UT>TL8 |
| 1.b | Flow | CD31 | 5 | 0.088 | 0.775 | **0.0003** | | 14188.02 | 11187.11 | 17188.93 | UT>TL8 |
| 1.b | Flow | CD200R | 5 | 0.087 | 0.820 | 0.2823 | | -38.00 | -167.19 | 91.19 | UT>TL8 |
| 1.b | Flow | SIRPα | 5 | 0.855 | 0.963 | 0.1659 | | 313.68 | -292.40 | 919.76 | UT>TL8 |
| 1.c | ELISA | TNFα | 4 | 0.710 | 0.940 | **0.0008** | | -11.96 | -14.57 | -9.36 | UT<TL8 |
| 1.c | ELISA | IL-12p40 | 4 | 0.996 | 0.986 | **0.0048** | | -6.05 | -8.46 | -3.64 | UT<TL8 |
| 1.c | ELISA | IL-1β | 6 | 0.398 | 0.932 | **0.0000** | | -8.81 | -9.30 | -8.33 | UT<TL8 |
| 2.a | qPCR | FcγR1a | 9 | 0.412 | 0.933 | 0.0002 | | 6.43 | 4.35 | 8.50 | UT<TL8 |
| 2.a | qPCR | FcγR2a | 9 | 0.848 | 0.936 | **0.0032** | | -28.76 | -43.41 | -14.11 | UT<TL8 |
| 2.a | qPCR | FcγR2b | 10 | 0.010 | 0.764 | | **0.0322** | 4.12 | 0.57 | inf | UT>TL8 |
| 2.a | qPCR | FcγR3a | 9 | 0.088 | 0.866 | 0.0834 | | 4.08 | -0.91 | 9.08 | UT<TL8 |
| 2.a | qPCR | FcεR1γ | 9 | 0.058 | 0.888 | **0.0017** | | -138.65 | -201.24 | -76.07 | UT<TL8 |
| 2.b | Western Blot | FcγR1a | 4 | 0.218 | 0.847 | 0.0478 | | 0.38 | 0.01 | 0.74 | UT<TL8 |
| 2.b | Western Blot | FcγR2a | 4 | 0.440 | 0.912 | **0.0308** | | -1.34 | -2.42 | -0.26 | UT<TL8 |
| 2.b | Western Blot | FcγR2b | 7 | 0.561 | 0.889 | **0.0423** | | 0.60 | 0.04 | 1.16 | UT>TL8 |
| 2.b | Western Blot | FcγR3a | 3 | 0.963 | 1.000 | **0.0090** | | -0.89 | -1.24 | -0.54 | UT<TL8 |
| 2.b | Westen Blot | Gamma chain | 6 | 0.069 | 0.805 | **0.0160** | | -10.45 | -17.60 | -3.31 | UT<TL8 |
| 2.c | Flow | FcγR1a | 7 | 0.377 | 0.819 | 0.3802 | | -70.43 | -499.34 | 358.49 | UT<TL8 |
| 2.c | Flow | FcγR2a | 7 | 0.974 | 0.955 | **0.0055** | | -786.00 | -1206.34 | -365.66 | UT<TL8 |
| 2.c | Flow | FcγR2b | 7 | 0.083 | 0.823 | **0.0327** | | 2346.07 | 319.86 | 4372.28 | UT>TL8 |
| 2.c | Flow | FcγR3a | 7 | 0.326 | 0.950 | 0.3976 | | -123.00 | -1003.62 | 757.62 | UT<TL8 |
| 3.a | Flow | FcγR1a | 7 | 0.156 | 0.821 | 0.1129 | | 650.29 | -285.76 | 1586.33 | UT<TL8 |
| 3.a | Flow | FcγR2a | 7 | 0.443 | 0.851 | **0.0014** | | -2486.57 | -3473.66 | -1499.48 | UT<TL8 |
| 3.a | Flow | FcγR3a | 7 | 0.048 | 0.735 | | 0.9843 | 137.00 | -inf | 470.00 | UT<TL8 |
| 3.a | Flow | FcγR2b | 7 | 0.140 | 0.775 | **0.0481** | | 1003.00 | 14.49 | 1991.51 | UT>TL8 |
| 3.b | Flow | CD86 | 12 | 0.978 | 0.935 | **0.0491** | | -255.83 | -510.12 | -1.55 | UT<TL8 |
| 3.b | Flow | CD63 | 12 | 0.000 | 0.568 | | **0.0019** | -103.17 | -inf | -64.50 | UT<TL8 |
| 3.b | Flow | CD69 | 12 | 0.013 | 0.724 | | 0.7407 | 23.00 | -inf | 133.00 | UT<TL8 |
| 3.b | Flow | CD11b | 12 | 0.732 | 0.969 | **0.0148** | | -504.83 | -867.65 | -142.01 | UT<TL8 |
| 4.a | Flow | CD31 | 5 | 0.989 | 0.999 | **0.0417** | | 1136.20 | 80.55 | 2191.85 | UT>TL8 |
| 4.a | Flow | CD163 | 4 | 0.934 | 0.985 | **0.0428** | | 2354.75 | 162.78 | 4546.72 | UT>TL8 |
| 4.a | Flow | CD86 | 4 | 0.385 | 0.894 | **0.0232** | | -5799.75 | -9960.60 | -1638.90 | UT<TL8 |
| 4.b | qPCR | BAFF | 6 | 0.426 | 0.856 | 0.4123 | | -0.23 | -2.17 | 1.72 | UT>TL8 |
| 4.b | qPCR | APRIL | 6 | 0.556 | 0.966 | **0.0491** | | 0.30 | 0.00 | 0.60 | UT>TL8 |
| 4.c | ELISA | TNFα | 7 | 0.527 | 0.960 | **0.0030** | | -2.86 | -4.20 | -1.52 | UT<TL8 |
| 4.c | ELISA | IL-12p40 | 6 | 0.246 | 0.888 | **0.0007** | | -5.12 | -6.76 | -3.48 | UT<TL8 |
| 4.c | ELISA | Granzyme B | 6 | 0.870 | 0.900 | **0.0244** | | -0.18 | -0.32 | -0.04 | UT<TL8 |
| 5.a | qPCR | CD69 | 7 | 0.091 | 0.901 | **0.0287** | | -22.18 | -40.56 | -3.81 | UT<TL8 |
| 5.a | qPCR | CD27 | 7 | 0.013 | 0.732 | | **0.0156** | 0.01 | 0.01 | Inf | UT>TL8 |
| 5.b | ELISA | Granzyme B | 6 | 0.859 | 0.947 | **0.0274** | | -3.66 | -6.62 | -0.71 | UT<TL8 |
| 5.b | ELISA | IFNγ | 3 | 0.939 | 0.999 | **0.0290** | | -8.01 | -13.91 | -2.11 | UT<TL8 |
| 6.a | Flow | CD182 | 3 | 0.319 | 0.878 | **0.0131** | | 11129.00 | 5770.97 | 16487.03 | UT>TL8 |
| 6.a | Flow | CD54 | 3 | 0.629 | 0.974 | **0.0087** | | -87131.00 | -121195.51 | -53066.49 | UT<TL8 |
| 6.a | Flow | CD95 | 3 | 0.898 | 0.997 | 0.0182 | | 2008.00 | 856.61 | 3159.39 | UT<TL8 |
| 6.b | Flow | FcγR1a | 7 | 0.690 | 0.984 | **0.0057** | | -533.00 | -820.36 | -245.64 | UT<TL8 |
| 6.b | Flow | FcγR2a | 7 | 0.274 | 0.876 | **0.0201** | | -686.29 | -1197.80 | -174.78 | UT<TL8 |
| 6.c | Phagocytosis | | 5 | 0.734 | 0.937 | **0.0171** | | -115.80 | -193.88 | -37.72 | UT<TL8 |

Part B: Results for statistical analysis of paired, 4 group analyses using the mixed effects model, with Hochberg correction for multiple testing. Confidence intervals are also shown.

| Figure | Measure | Marker | Sample size | Label | Difference ($log_2$value) | p-value (Mixed-effect model) | 95% Confidence Interval (lower,upper) | Hochberg p-value |
|---|---|---|---|---|---|---|---|---|
| 2.d | ELISA | TNFα | 3 | Combination vs TL8-506 | 0.1987 | 0.0007 | (0.12,0.27) | 0.0014 |
| | | | | Combination vs Untreated | 3.9966 | 0.0000 | (3.92,4.07) | 0.0000 |
| | | | | Combination vs dIgG | 3.9966 | 0.0000 | (3.92,4.07) | 0.0000 |
| | | | | TL8-506 vs Untreated | 3.7978 | 0.0000 | (3.72,3.87) | 0.0000 |
| | | | | Untreated vs dIgG | 0.0000 | 1.0000 | (-0.08,0.08) | 1.0000 |
| 5.c | Flow | Granzyme B | 7 | Combination vs Obinutuzumab | 0.4875 | 0.0009 | (0.23,0.74) | 0.0035 |
| | | | | Combination vs TL8-506 | -0.0377 | 0.7617 | (-0.29,0.22) | 0.8815 |
| | | | | Combination vs Untreated | 0.4690 | 0.0012 | (0.21,0.73) | 0.0037 |
| | | | | Obinutuzumab vs Untreated | -0.0185 | 0.8815 | (-0.28,0.24) | 0.8815 |
| | | | | TL8-506 vs Untreated | 0.5067 | 0.0006 | (0.25,0.76) | 0.0031 |
| 5.c | Flow | IFNγ | 7 | Combination vs Obinutuzumab | 2.3633 | 0.0042 | (0.87,3.86) | 0.0183 |
| | | | | Combination vs TL8-506 | 0.9825 | 0.1811 | (-0.51,2.48) | 0.3622 |
| | | | | Combination vs Untreated | 2.7680 | 0.0046 | (1,4.54) | 0.0183 |
| | | | | Obinutuzumab vs Untreated | 0.4047 | 0.6334 | (-1.37,2.18) | 0.6334 |
| | | | | TL8-506 vs Untreated | 1.7855 | 0.0485 | (0.01,3.56) | 0.1454 |
| 5.d | ADCC (fllow) | NA | 6 | Combination vs Obinutuzumab | -0.3830 | 0.0135 | (-0.67,-0.09) | 0.0270 |
| | | | | Combination vs TL8-506 | -1.3744 | 0.0000 | (-1.67,-1.08) | 0.0000 |
| | | | | Combination vs Untreated | -1.3941 | 0.0000 | (-1.69,-1.1) | 0.0000 |
| | | | | Obinutuzumab vs Untreated | -1.0110 | 0.0000 | (-1.3,-0.72) | 0.0000 |
| | | | | TL8-506 vs Untreated | -0.0197 | 0.8877 | (-0.31,0.27) | 0.8877 |

**Supplementary Table S3.** Summary of statistical analyses. For Part A, p-values in bold are counted as significant; others either did not meet the 0.05 level or were opposite to our *a-priori* predictions and thus not significant by rule.

| Group Size | Age | | | | Sex | | WBC Count (thousand per µL) | | | | Treatment Status | |
|---|---|---|---|---|---|---|---|---|---|---|---|---|
| | Mean | Median | Minimum | Maximum | Female | Male | Mean | Median | Minimum | Maximum | naïve | previously treated |
| 73 | 70 | 67 | 60 | 93 | 36 | 37 | 15.49 | 14.03 | 3.98 | 34.08 | 73 | 0 |

| Treatment Status | | Study Inclusion | |
|---|---|---|---|
| naïve | previously treated | Inclusion Criteria | Exclusion Criteria |
| 73 | 0 | *Age ≥ 18 years* | *None* |

**Supplementary Table S4.** Basic demographics of patients. All samples were de-identified prior to use. Disease stage, IGHV mutational status and cytogenetics were not available.

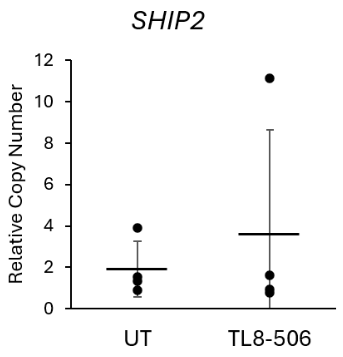


**Supplementary Figure S1.** TLR8 agonist treatment did not lead to changes in SHIP2 expression. Monocytes were isolated and treated as in Figure 1, then SHIP2 transcripts measured using qPCR.

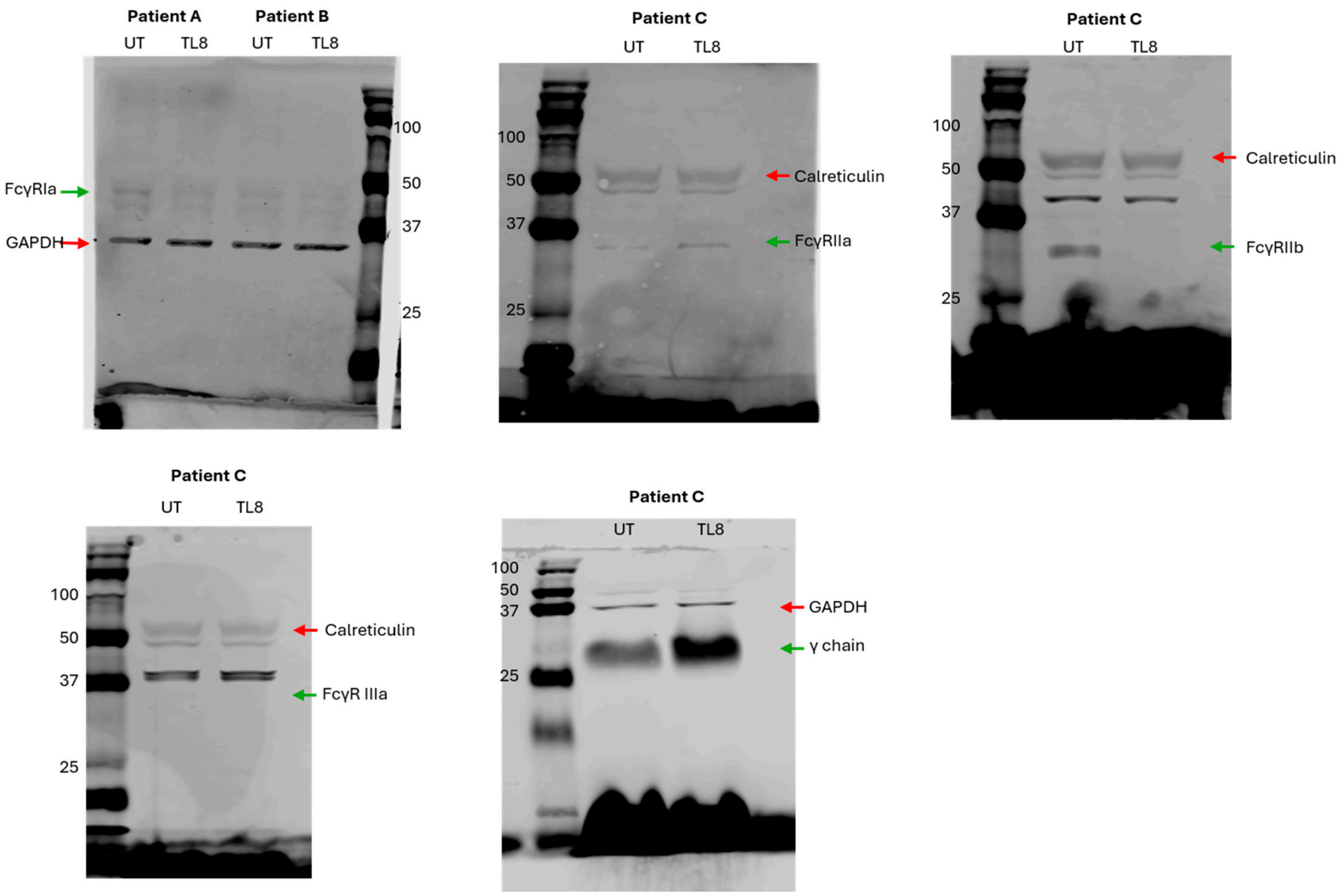


**Supplementary Figure S2.** Whole-membrane scans for immunoblots shown in Figure 2. Numbers next to blots denote sizes in kDa within the molecular-weight ladder. A 15% gel was used for the γ-chain (bottom right panel), while 10% gels were used for all others.

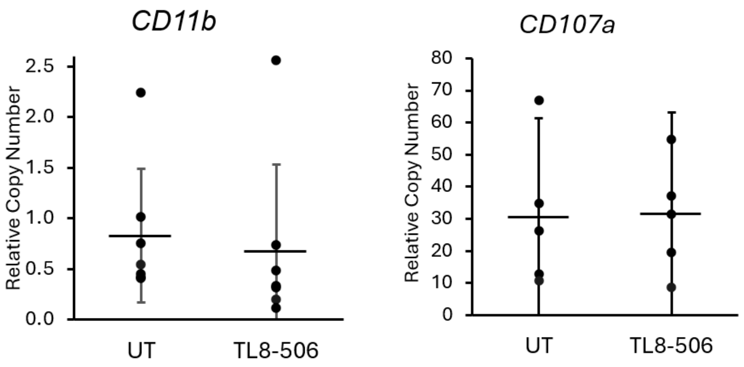


**Supplementary Figure S3.** TLR8 agonist treatment does not lead to transcriptional changes in *CD11b* nor *CD107a*. NK cells were isolated and treated as in Figure 5, then *CD11b* and *CD107a* measured using qPCR.

| A | Patient 1 | Patient 2 |
| --- | --- | --- |
| UT | 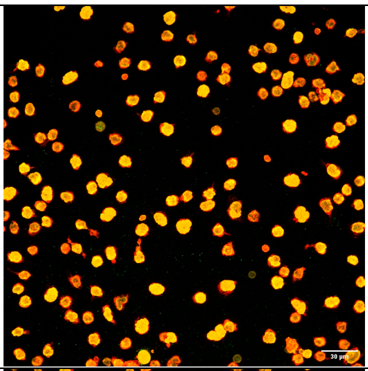 | 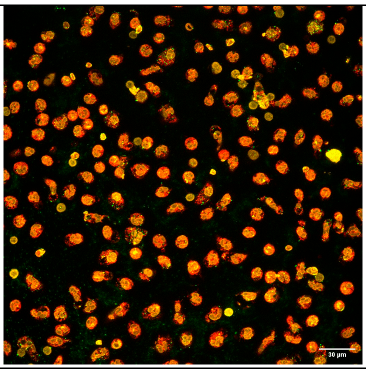 |
| DMSO | 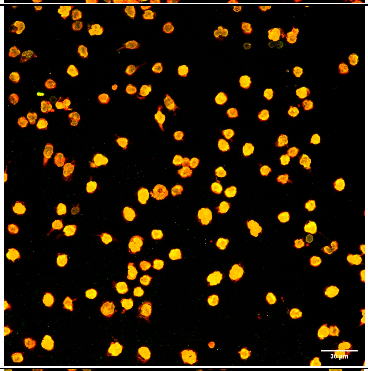 | 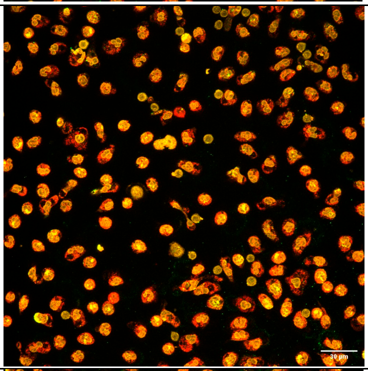 |
| TL8-506 | 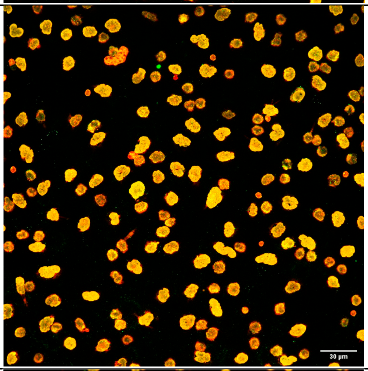 | 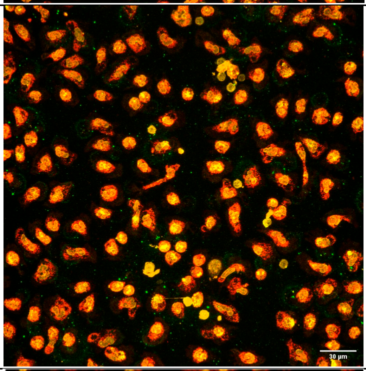 |
| PMA | 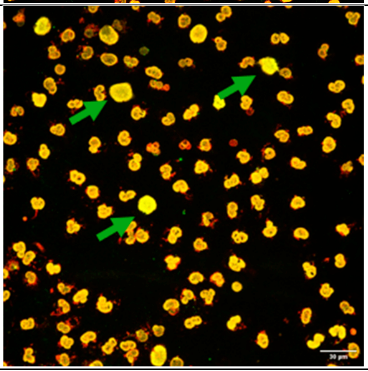 | 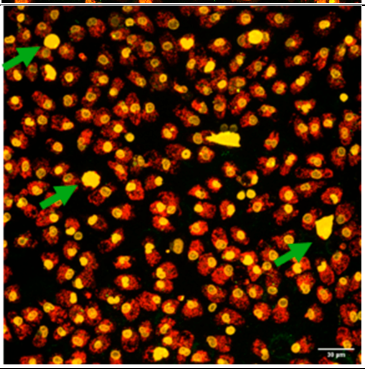 |

*(continued on next page)*

<table>
<tr><th>B</th><th>Patient 1</th><th>Patient 2</th></tr>
<tr><td>Sytox Reads</td><td>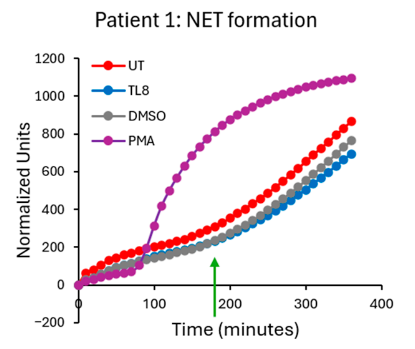
</td><td>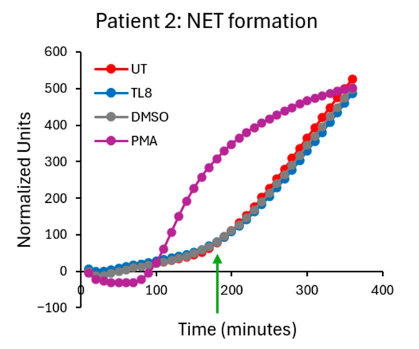
</td></tr>
</table>

**Supplementary Figure S4.** Treatment with TL8-506 does not induce NET formation. A) Isolated neutrophils from CLL patients were plated in 8-well culture slides and left untreated (UT) or treated with TL8-506 1 μM, DMSO or PMA 50 nM for 3 hours. Cells were then fixed and stained for detection of myeloperoxidase (Alexa Flour 488; green), double-stranded DNA (Alexa Flour 555, yellow) and membrane (WGA – Alexa Flour 647, red). **B)** Isolated neutrophils from the same patients were tested for NET formation / cell death by kinetic Sytox Green fluorescence detection using a plate reader as described in Materials and Methods. Green arrows indicate "Cloud cells," a sign of early-stage NET formation. Scale bars in lower right panels of images represent 30 microns.

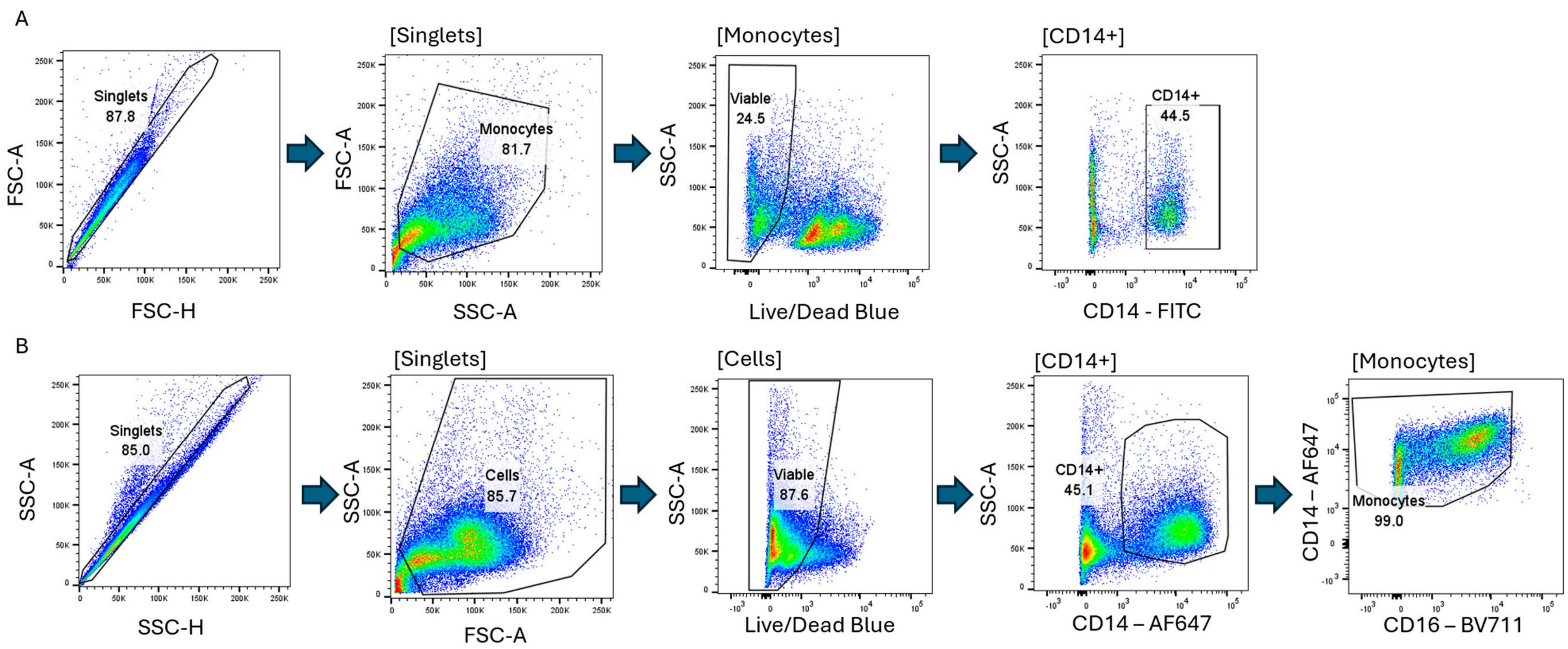


**Supplementary Figure S5.** Gating strategy followed for isolated monocytes to determine expression of checkpoints and FcγR. Experiment was done with and without TL8-506 for 48 hours. Initial purity for the presented samples immediately after isolation was approximately 90%. After elimination of doublets and dead cells, monocytes were identified by CD14+ expression. Negative cells might be monocytes entering the early phases of apoptosis or naturally lost in culture conditions (PMID: 9317115; DOI: 10.1016/j.jim.2011.03.006). A) Gating prior to measurement of checkpoints. B) Gating prior to measurement of FcγR.

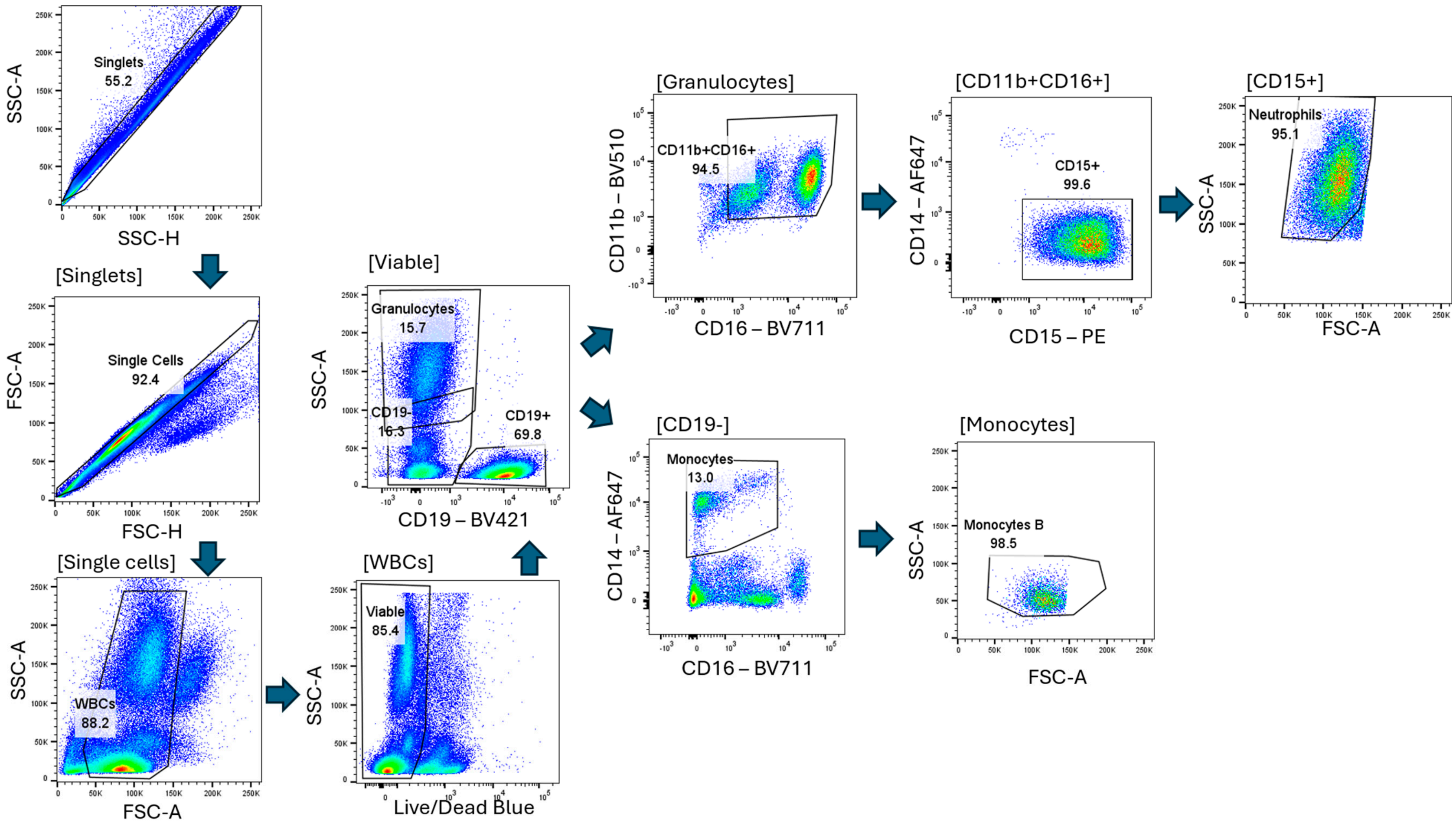


**Supplementary Figure S6.** Gating strategy to identify monocytes and neutrophils in WBC cultures. After gating out doublets, dead and B cells (CD19$^{+}$), the granulocyte and CD19$^{-}$ populations were followed to either identify neutrophils (CD11b$^{+}$, CD16$^{+}$, CD15$^{+}$ and CD14$^{-}$) and monocytes (CD56$^{-}$, CD14$^{+}$).

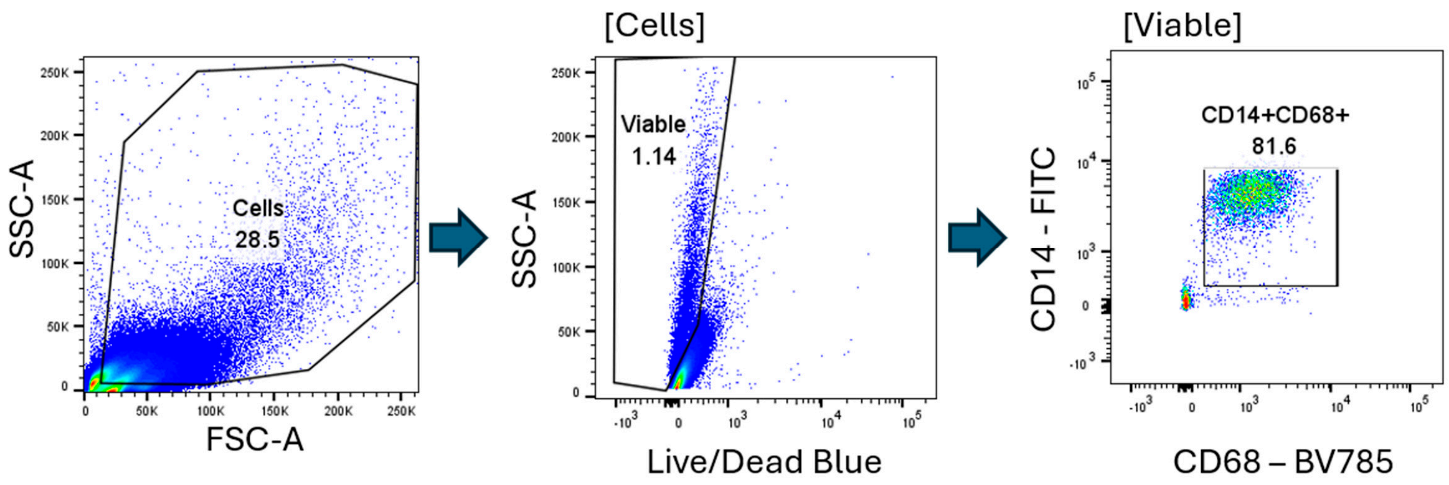


**Supplementary Figure S7.** Gating strategy to identify NLCs in WBC long-term cultures. Viable cells expressing CD14 and CD68 were selected. NLCs are high in complexity and size, so doublet elimination was not done.

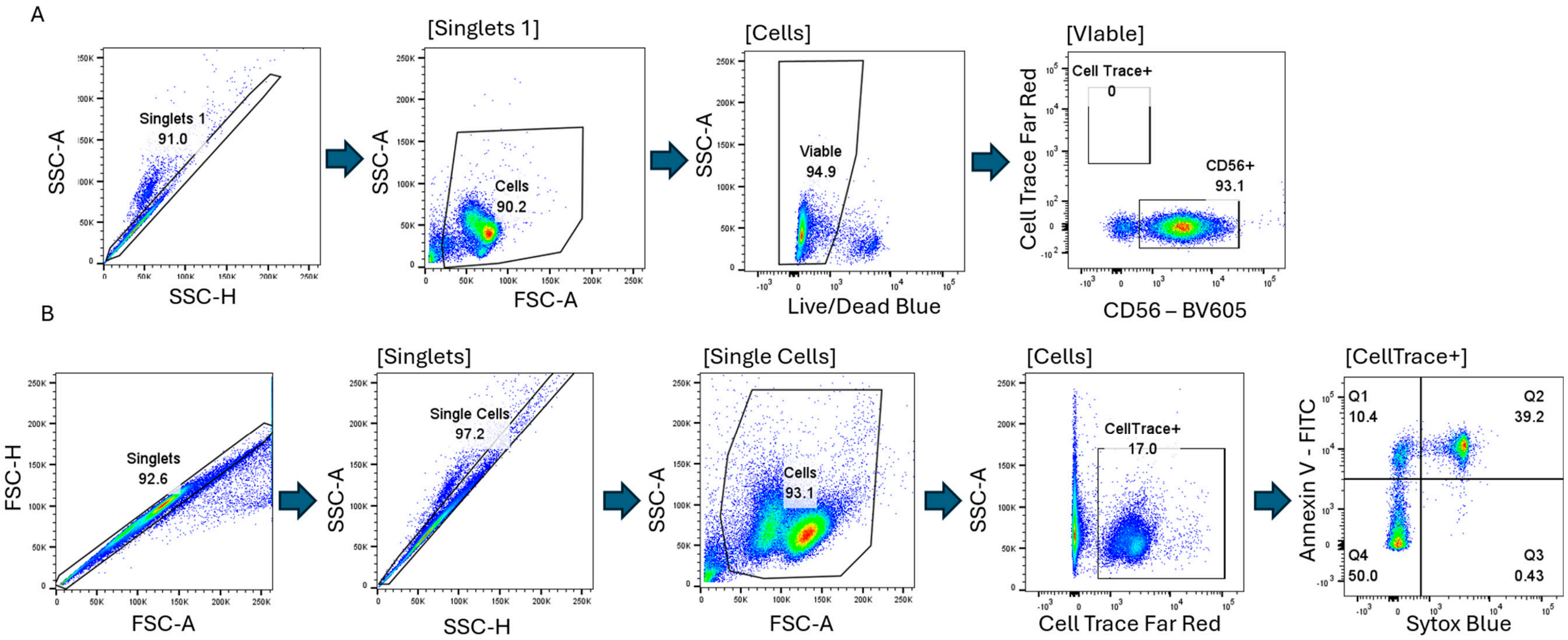


**Supplementary Figure S8.** Gating strategy for NK-cell and target cell identification in the NK-cell cytotoxicity assays. A) Doublets, debris and dead cells were eliminated, then NK cells identified by CD56 expression. B) For the cytotoxicity assay, target cells were pre-labeled with Cell Trace Far Red, then assessed via Annexin V / Sytox staining.

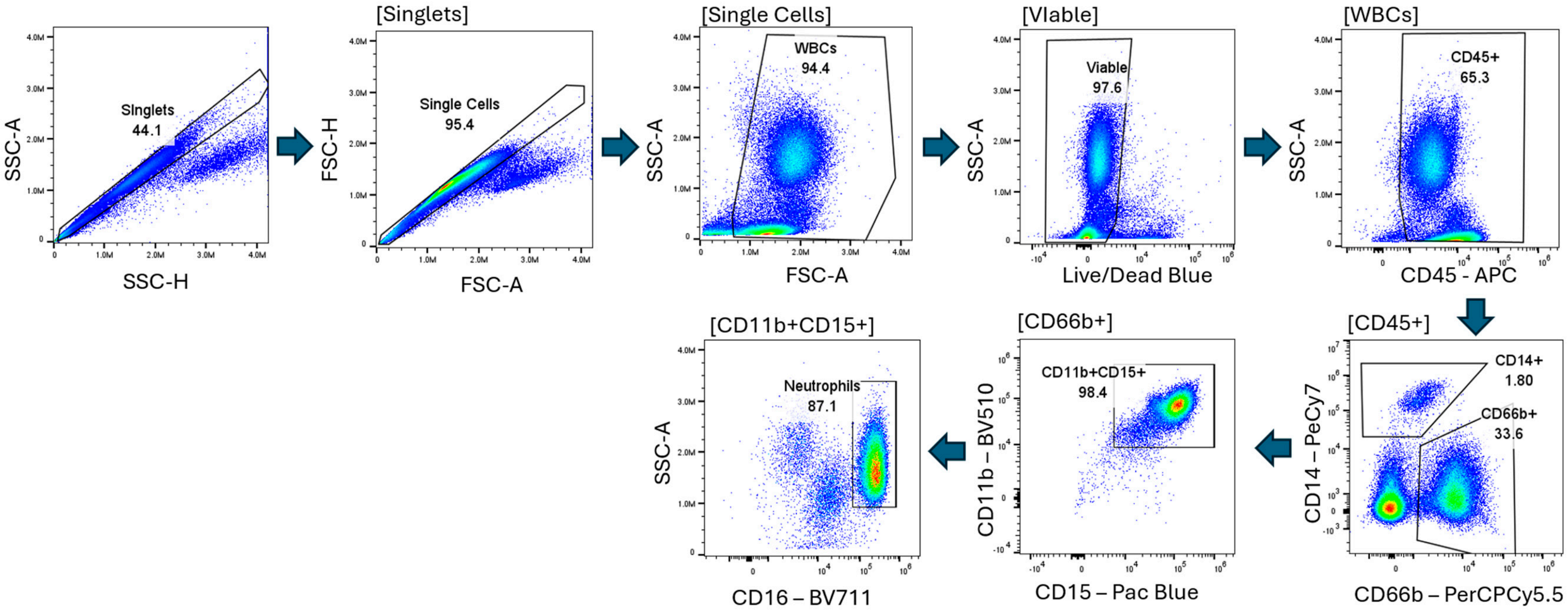


**Supplementary Figure S9.** Gating strategy to identify neutrophils in WBCs following 48-hour treatments with TL8-506. Doublets, debris and dead cells were gated out, then gating done for $CD45^+$, $CD66b^+$, $CD11b^+$, $CD15^+$ and $CD16^+$.
